# Distribution of Mechanical Properties in Poly(ethylene oxide)/silica Nanocomposites via Atomistic Simulations: From the Glassy to the Liquid State

**DOI:** 10.1021/acs.macromol.4c00537

**Published:** 2024-04-29

**Authors:** Hilal Reda, Ioannis Tanis, Vagelis Harmandaris

**Affiliations:** † Computation-based Science and Technology Research Center, The Cyprus Institute, Nicosia 2121, Cyprus; ‡ Department of Mathematics and Applied Mathematics, University of Crete, Heraklion GR-71110, Greece; § Institute of Applied and Computational Mathematics, Foundation for Research and Technology - Hellas, Heraklion GR-71110, Greece

## Abstract

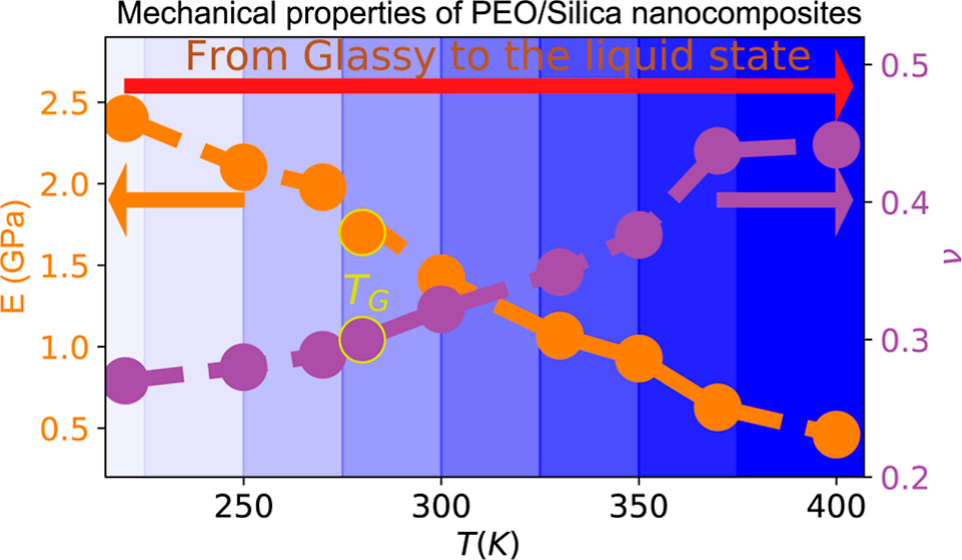

Polymer nanocomposites exhibit a heterogeneous mechanical behavior that is strongly dependent on the interaction between the polymer matrix and the nanofiller. Here, we provide a detailed investigation of the mechanical response of model polymer nanocomposites under deformation, across a range of temperatures, from the glassy regime to the liquid one, via atomistic molecular dynamics simulations. We study the poly(ethylene oxide) matrix with silica nanoparticles (PEO/SiO_2_) as a model polymer nanocomposite system with attractive polymer/nanofiller interactions. Probing the properties of polymer chains at the molecular level reveals that the effective mass density of the matrix and interphase regions changes during deformation. This decrease in density is much more pronounced in the glassy state. We focus on factors that govern the mechanical response of PEO/SiO_2_ systems by investigating the distribution of the (local) mechanical properties, focusing on the polymer/nanofiller interphase and matrix regions. As expected when heating the system, a decrease in Young’s modulus is observed, accompanied by an increase in Poisson’s ratio. The observed differences regarding the rigidity between the interphase and the matrix region decrease as the temperature rises; at temperatures well above the glass-transition temperature, the rigidity of the interphase approaches the matrix one. To describe the nonlinear viscoelastic behavior of polymer chains, the elastic modulus of the PEO/SiO_2_ systems is further calculated as a function of the strain for the entire nanocomposite, as well as the interphase and matrix regions. The elastic modulus drops dramatically with increasing strain for both the matrix and the interphase, especially in the small-deformation regime. We also shed light on characteristic structural and dynamic attributes during deformation. Specifically, we examine the rearrangement behavior as well as the segmental and center-of-mass dynamics of polymer chains during deformation by probing the mobility of polymer chains in both axial and radial motions under deformation. The behavior of the polymer motion in the axial direction is dominated by the deformation, particularly at the interphase, whereas a more pronounced effect of the temperature is observed in the radial directions for both the interphase and matrix regions.

## 1 Introduction

Polymer nanocomposites (PNCs) have gained considerable attention over the last few decades, as the addition of nanoparticles in a polymer may drastically alter the properties of the matrix, and in particular its mechanical behavior.^1−10^ For example, an enhancement of the polymer stiffness and the wear resistance in tire manufacturing, by adding nanofillers in a polymer matrix, has been reported.^11−14^ A common characteristic of all PNCs is that their mechanical properties change dramatically within a relevant short, compared to other systems like metals, range of temperature, due to their viscoelastic behavior.^15−22^

Poly(ethylene oxide)/silica nanocomposites have demonstrated significant potential in the domains of engineering and nano(bio)technology, rendering them a subject of considerable interest for ongoing research and development endeavors.^3,23,24^ Poly(ethylene oxide) (PEO) is a multifaceted polymer that exhibits a wide range of technical uses. PEO is a nonionic, water-soluble, semicrystalline polymer that is widely utilized in various applications mainly due to its biocompatibility, hydrophilicity, high degree of crystallinity, and its ability for ion conduction.^25−27^ Among the various nanoadditives, silica (SiO_2_) nanoparticles have been utilized to develop PEO/SiO_2_ nanocomposites. In this system, attractive interactions exist between PEO and the silica surfaces, mainly due to the formation of hydrogen bonds, which leads polymer chains to adsorb onto the nanofiller surface stabilizing the system.^28−30^

Such changes in the mechanical response of the PNCs are typically described as heat distortion,^31,32^ taking place over a wide temperature range, which, for a variety of polymeric materials and applications, lies between 300 and 450 K.^33^ This temperature range includes, for several polymer-based nanostructured systems, the transition from the glass state toward the liquid (viscoelastic) one.

The temperature dependence of the mechanical behavior of PNCs has been extensively studied through experiments in the recent past. For example, Kontou and Anthoulis have used different techniques, including scanning electron microscopy, differential scanning calorimetry, dynamic mechanical analysis, and tensile testing, to probe the mechanical properties of a series of polystyrene (PS)/silica nanocomposites at three different temperatures.^34^ The mechanical enhancement was manifested through the response of tensile stress–strain, while the temperature effect was found to shift the response from brittle at 293 K to rubbery behavior at 358 K. Using a dynamic mechanical analyzer, Liu et al.^35^ have shown that the Young’s modulus of a thermoset SMP (shape memory polymer) epoxy system reinforced with 20 wt % SiC (silicon carbide) at *T* = 299 K is approximately 2 orders of magnitude higher than that at *T* = 391 K and the Young’s modulus for the nanocomposite is higher than that of the SMP resin.

Unfortunately, accurate experimental investigation of parameters related to physical mechanisms on the atomic scale and consideration of temperature effects are generally challenging, time-consuming, and cost-intensive. Moreover, experimental investigations of the mechanical properties of PNCs within the heat distortion temperature range are rather challenging due to the complex and spatially heterogeneous mechanical response of PNCs and the random dispersion of nanoparticles within the polymer matrix.^36,37^ Noteworthily, semiempirical approaches, to predict the effect of temperature on global mechanical properties, have been proposed during the last few decades.^38−40^ Such works are typically based on robust physics-based models for the prediction of the rigidity modulus for a wide range of temperatures below and above the glass-transition temperature, *T*_g_, and frequencies/strain rates. Analysis of the parameters (e.g., the size and shape of the nanoparticle, the agglomeration, as well as the interaction between the matrix and nanofiller) allowed the introduction of empirical equations to consider the time/temperature dependence in the model.^41−43^

In addition to experiments, several theoretical models have been developed to study the temperature dependence of mechanical properties in PNCs. For example, Richeton et al.^44^ have developed a theoretical model for the elastic Young’s modulus, which takes into account the effect of temperature. The basis of this work is the statistical model for modulus dependence on temperature, which was developed by Mahieux and Reifsnider;^32,45^ in the latter, authors used Weibull moduli (mi) to represent the activation bond breakage energy as a function of temperature. The temperature dependence of Young’s modulus has been further examined by theoretical models, such as the semiempirical Vogel–Fulcher–Tammann equation (VFT) and the mode coupling theory (MCT).^46−49^ These models suggest that the principle of temperature superposition remains modestly above the glass-transition temperature, in the viscoelastic region. It is also well known that, in addition to temperature, strain rate, and thermal history (e.g., cooling rate), it could strongly affect the mechanical response of polymer chains and in particular their shear modulus and the failure mechanism.^50,51^ The effect of temperature variation on the thermo-mechanical properties of PNCs, which contain spherical nanoparticles, was further recently investigated using thermo-micromechanical models.^52^

Molecular simulations can shed light on the microscopic mechanisms that affect mechanical reinforcement in PNCs, at the atomic/molecular scale, by controlling and tuning the rather complex set of parameters that can affect the mechanical behavior of the hybrid materials. Such parameters are associated with the type of nanofillers (e.g., their size, shape, and morphology), the polymer matrix (e.g., molecular weight, chemistry, and topology), and the concentration (loading) of nanoparticles and the polymer–nanoparticle interaction (attractive, neutral, or repulsive). All of the above factors determine the dispersion state of the nanofillers and the properties of the entire hybrid system. On these grounds, and considering that in model systems the above system characteristics can be relatively accurately controlled, it is not surprising that the mechanical properties of polymer matrices embedded with nanoparticles have been studied extensively in the past years by molecular simulations using atomistic and coarse-grained models.^53−65^ The effects of nanoparticle size and properties, polymer–nanofiller interactions, chain cross-links and entanglements and the temperature on the stress–strain behavior, failure mechanism, and mechanical reinforcement of PNCs are comprehensively investigated using molecular simulations.^37,66−69^

Another important phenomenon that has been investigated during the last decades concerns the nonlinear dependence of the Young’s modulus on strain, derived from the stress–strain curve; a behavior that is analogous to the nonlinear dynamic viscoelastic one of the storage modulus, typically called the Payne effect.^69−72^ The latter is of great significance in practical applications such as the rolling and sliding resistance of tires.^73,74^ Moreover, the Young’s modulus and Poisson’s ratio of an amorphous linear polyethylene-like polymer have also been examined via molecular simulation approaches under different temperatures; the simulation predictions show good agreement with experimental findings for the temperature and strain rate dependencies of stress–strain curves.^75^

Typically, experimental and simulation studies of mechanical properties in PNCs consider the effect of temperature on the global (average) mechanical properties of glassy PNCs without dealing with the role of temperature on their (spatial) heterogeneous mechanical response. The distribution of local mechanical properties of polymeric nanocomposites has been investigated using Monte Carlo simulations, at equilibrium, for a wide range of temperatures using generic bead spring models.^7^ As expected, Young’s modulus decreased with increasing temperature, reflecting structural changes in the polymer matrix.

The works presented above unveiled a number of major challenges in providing a fundamental understanding of the mechanical behavior of PNCs, including the influence of temperature on the local mechanical properties of the subdomains, mainly the interphase and matrix regions, during the increasing tensile test. It is also of great importance to further elucidate the mechanism(s) of the effect of temperature on the mechanical behavior of heterogeneous polymer-based hybrid materials. Moreover, although some studies have addressed the origin of the mechanical reinforcement observed in PNCs,^76−81^ to our knowledge, the coupling between spatial variations of the mechanical properties of polymer chains in nanocomposites as a function of temperature, in the transition from the glassy state to the liquid (or rubbery) state, and the mobility of chains under deformation, has been poorly investigated. The latter is particularly interesting, as both elastic moduli and load transfer through the nanofillers are greatly influenced by temperature, especially when the latter is close to or above the glass-transition temperature (*T*_g_) of the polymer matrix.^82,83^

In this work, we investigate the mechanical properties of PNCs under deformation, via detailed atomistic simulations, across a broad range of temperatures from the glassy to the rubbery regime, focusing on the variation of the spatial distribution of stress and strain fields at the polymer/nanofiller interphase and matrix regions. We study poly(ethylene oxide)/silica, PEO/SiO_2_, systems as a model PNC with a moderately attractive polymer/nanoparticle interaction. The equilibrium structural and dynamic properties of PEO/SiO_2_ systems have been extensively examined by both simulations^28,62,84−86^ and experiments.^28,85,87,88^ Our methodology for probing the distribution of local mechanical properties is based on a recent multiscale computational approach computing effective (per atom) stress and strain fields within atomistic model PNCs under an applied external field.^65,89^

In the rest of the paper, we provide in Section 2 details about the model systems and the simulations focusing on the deformation process and the way we compute the local stress and strain fields. Then, in Section 3, we investigate the structural (density) heterogeneities of the PEO/SiO_2_ model systems at equilibrium and under deformation. Results concerning the average, as well as the distribution of, mechanical properties of hybrid systems across a range of temperatures are presented and discussed in Sections 4 and 5. In the same sections, on the basis of the global and local stress–strain behavior, the elastic moduli are obtained as a function of strain. A detailed investigation of the mobility of polymer chains as a function of the deformation for the interphase and matrix regions and for different temperatures is presented in Section 6. Finally, in Section 7, we summarize our findings and discuss current and future challenges.

## 2 Model and Simulation Details

**Figure 1:**
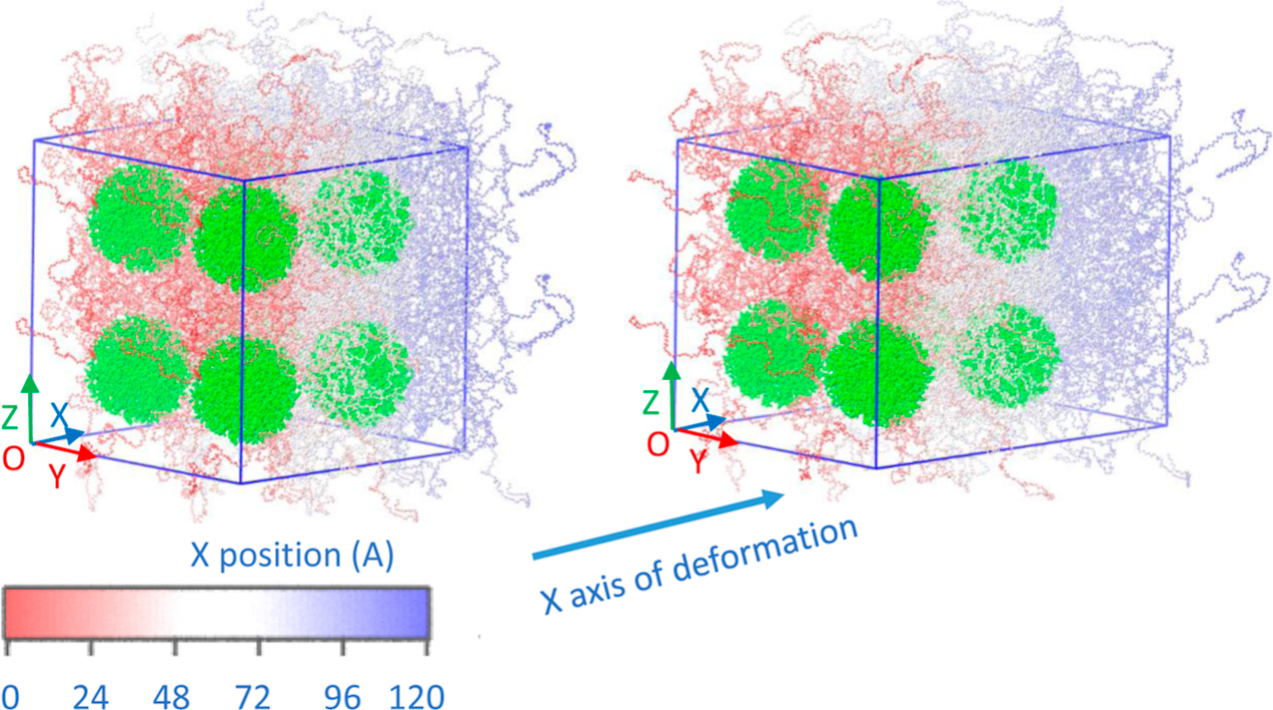
Typical snapshots of the initial equilibrium (left) and deformation in the *x* direction (ϵ = 0.3) configurations (right) of the PEO/SiO_2_ model systems at *T* = 400 K. The different colors in the representation of the polymer chains denote their relative positions along the *X* direction, with respect the origin of the Cartesian coordinate system, O.

Atomistic MD simulations are performed for model PEO/SiO_2_ nanocomposites with 33 wt % (12.7 vol %, ) silica nanoparticles, that is a typical volume nanoparticle fraction used in PEO/SiO_2_ hybrids.^28^ All force field parameters for PEO and silica are provided in Section S1 in the Supporting Information. The Lorentz–Berthelot mixing rule was used for the calculation of nonbonded interactions between PEO atoms and silica atoms. The time step in all MD simulation runs was 1 fs, and each elongation run was of duration of at least 10 ns. Coulombic interactions were evaluated using the particle mesh Ewald method, while a cutoff point of 1 nm was used to calculate van der Waals interactions. The polymer matrix consists of 48 unentangled PEO chains of 50 monomers each (the molecular weight is about 2.2 kDa), terminated with methyl groups, while a silica nanoparticle, with an almost spherical shape, of radius ≈2.0 nm, was dispersed in the PEO matrix. The PEO chains correspond to unentangled, Rouse-like, polymer chains well above the oligomeric regime. PEO/silica systems with PEO of similar molecular weight have been extensively studied experimentally in the recent past.^90^ On the basis of these works and given the limitations of atomistic simulations with respect to high-molecular weight chains, in the current work, we focus on investigating the role of temperature and of the polymer/nanofiller interaction on the global mechanical reinforcement of unentangled systems. The potential coupling between mechanical behavior and entanglements would require systematic coarse-graining methodologies. Snapshots of the model PEO/SiO_2_ systems before and after 0.3 deformation are shown in Figure 1. We should note that PEO/SiO_2_ is a typical example of well-dispersed nanofillers due to the attractive polymer/nanofiller interaction. The silica NP was created following a procedure that generates amorphous bulk silica and has been described by a fully flexible all-atom force field.^91^ A methodology involving a melt-quenching process for preparing amorphous silica given by Vollmayr et al. is used as a precursor to nanoparticle preparation.^92^

First, we prepared well-equilibrated configurations of a small system, containing a single silica nanoparticle, at 400 K, well above the glass-transition temperature of PEO. For this, we first inserted the polymer chains into a large simulation box containing a SiO_2_ nanoparticle. After energy minimization, a several microsecond-long *NPT* equilibration run was performed. To ensure full equilibration, its length (after the density had reached its correct value) was several times longer than the relaxation time of the end-to-end vector of the polymer chains. After generating equilibrated structures, we multiply the simulation boxes to obtain systems containing several nanoparticles and further equilibrate the sample at a high temperature (*T* = 400 K). A series of runs was conducted at temperatures *T* = 220, 250, 270, 300, 330, 350, 370, and 400 K. The size of the simulation box changes from 59 Å at 400 K to 57 Å at 220 K; this leads to a small decrease in volume percentage. Note that the glassy transition temperature (*T*_g_) of the model PEO/SiO_2_ systems is around 280 K,^28,86^ while for PEO bulk is around *T* = 250 K (for more information about the determination of glassy transition temperature, see Section S5 in the Supporting Information).^93^

The samples were then cooled to the desired temperatures at a rate of 1–10 K/ns that is lower than typical cooling rates reported in simulation literature. However, we should note that in general, the cooling rates used in atomistic simulations are very rapid (of the order of 1 K/ns) compared to the experimental quenching rate that is approximately 1 K/s.^86^ Given the semiempirical observation that *T*_g_ is strongly affected by the cooling rate of approximately 3–5 degrees per decade, we expect differences between 30 and 50 K. Rapid cooling can result in finer microstructures, and the atoms within the molecular chains have less time to arrange themselves into a more ordered structure, which often leads to enhancing mechanical properties and making the material more brittle. Last, before applying the deformation simulations, we perform additional short MD runs for thermal and local structure equilibration; for more information about the thermodynamics equilibrium, see Section S2 in the Supporting Information. All model atomistic PEO/SiO_2_ systems considered in the present work include 8 SiO_2_ nanoparticles and 384 PEO chains, whereas the size of the simulation cubic box at equilibrium, in each direction, varies from 5.7 nm at 220 K to 5.9 nm at 400 K. More details on the molecular model of PEO and silica nanoparticles can be found elsewhere.^28,65^ Finally, during the discussion of the results, we also refer to data on the mechanical properties of the same system deep in the glassy regime (*T* = 150 K) taken from our previous work.^65,81^

After the preparation of the equilibrated PEO/SiO_2_ model systems, the latter were uniaxially deformed with constant strain rate . Because the overall mechanical behavior of the nanocomposite is expected to be isotropic due to the presence of approximately spherical inclusions (nanofillers), the rigidity matrix and the values of the Young’s modulus and Poisson’s ratio can be determined from a single axial tensile test across only one direction. We should note here that comparing atomistic molecular dynamics simulation results with experimental measurements is not a trivial issue due to the different values of strain rates considered; usually, tensile experiments are performed under low strain rates within the range of 10° s^–1^,^94−96^ whereas MD simulations involve much higher strain rates, greater than 10^6^ s^–1^.^97−99^

The deformation is applied for strain values up to 0.8, but here we focus mainly on the linear-like elastic regime, mainly for strains up to 0.1. Tensile deformations are performed under a specific statistical ensemble depending on the direction of deformation, that is, assuming deformation in the *x* direction. The deformations are performed under the *NTL*_*x*_σ_*yy*_σ_*zz*_ ensemble, i.e., constant temperature and normal stresses in *y* and *z* directions are imposed, for a given deformation in *x* direction, using the Nose–Hoover thermostat and Parrinello–Rahman barostat, respectively (we do not impose any cubic symmetry during the deformation). The uniaxial deformation of the box respects the periodic boundary condition along the axis of deformation; that is, each time the size or shape of the box is changed, the atom positions are remapped to the new box.

Here, we focus on the temperature dependence of a given load during an incremental tensile test. Concerning the dispersion of the nanoparticles in the polymer matrix, the model PEO/SiO_2_ systems correspond to a well-dispersed scenario, in which the silica nanoparticles are in a simple cubic-like arrangement within the polymer matrix, i.e., there is no aggregation of the nanoparticles. This is achieved by generating systems comprising a single nanoparticle embedded in the polymer matrix and replicating it twice along each Cartesian direction to obtain the final model PNCs, as shown in Figure 1. The methodology developed to evaluate mechanical properties, such as Young’s modulus and Poisson’s ratio, of the atomistic systems was inspired by continuum mechanics, utilized for the characterization of materials’ properties. In continuum mechanics, Young’s modulus and Poisson’s ratio are measured from the simple tension test by applying incremental strain at a constant strain rate. The same concept was extended and applied to the atomistic structures by performing MD simulations in the *NTL*_*x*_σ_*yy*_σ_*zz*_ ensemble, to allow variations in the size and shape of the simulation box during the deformations. To investigate the local distribution of the mechanical behavior of the model PEO/SiO_2_ systems, we need to probe the stress and strain field at the atomic level. To do so, we use a per atom calculation of stress and strain under an imposed global strain. Stress per atom can be directly computed for each atom *i*, σ_*i*_, via the atomic Virial formalism. Concerning the local strain, here we use a recently proposed methodology to directly probe the strain field in PNCs at the atomic level.^97,100^ First, the deformation gradient for each atom is calculated by solving a minimization problem related to the position of the atom of interest in its neighboring atoms within the cutoff radius *r*_cut_, allowing us to probe the distribution of the strain fields in the atomistic model using the definition of the Lagrange Green strain tensor with respect to the reference coordinates. More details on the extraction of mechanical properties through atomistic MD simulations can be found in Section S3 in the Supporting Information and in our previous work.^65,81,89,97^

## 3 Structural Properties of PEO/SiO_2_ Nanocomposites

### 3.1 Density Heterogeneities in PEO/SiO_2_ Nanocomposites at Equilibrium

**Figure 2:**
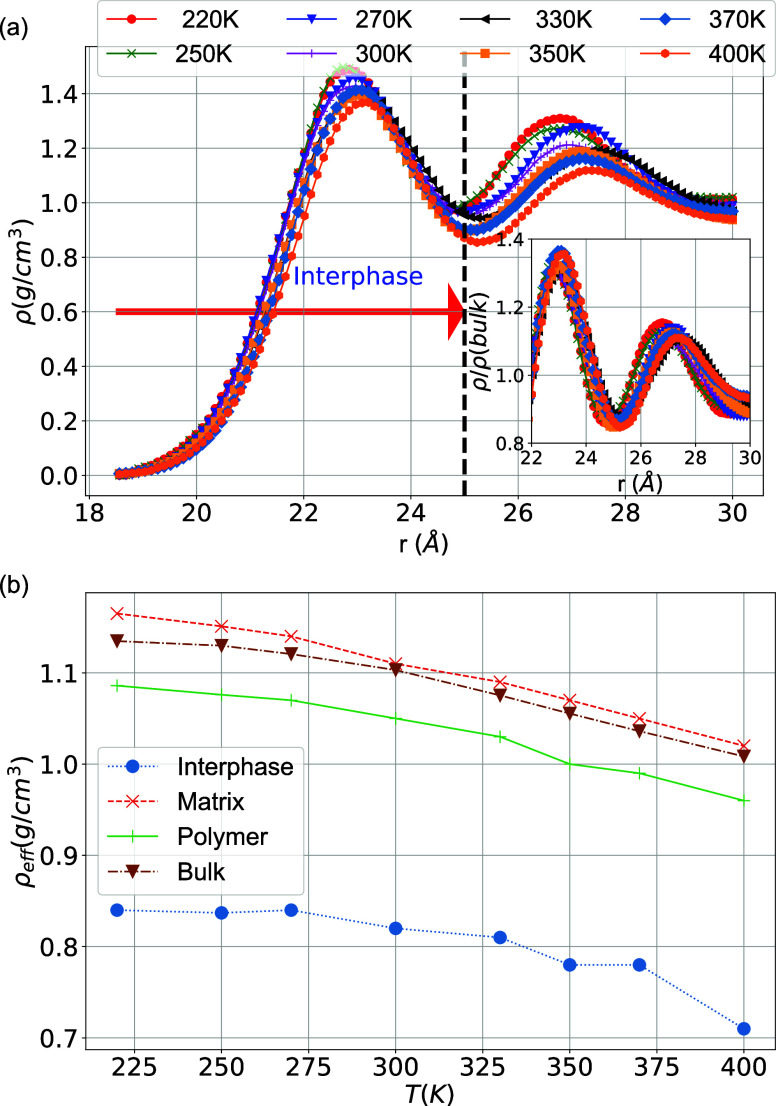
(a) Interfacial atomic density profiles of PEO chains, ρ(*r*), as a function of the distance from the center of the SiO_2_ nanoparticle. The interfacial density profiles were calculated by measuring the density of PEO atoms in thin spherical shells of thickness 0.06 Å around the silica NP. The red arrow indicates the thickness of the interphase. (Inset) Normalized density on the bulk values ρ/ρ_bulk_ focusing on the two peaks in the density profile and (b) effective mass density for the interphase, matrix, and polymer region as a function of temperature. For reference, we add the density in the bulk PEO system for different temperatures.

The first part of our analysis concerns the identification of the PEO/SiO_2_ interphase (denoted also as interface in literature) for different temperatures. In general, it is well known that the width of the polymer/nanoparticle interphase depends on the property under study.^55,101−103^ Here, we investigate the mechanical properties of the hybrid systems by computing the stress and strain fields at the local (atomic) level. When a reference atom is assumed, the per-atom values of local stress and strain fields are expected to depend strongly on its neighboring atoms. Therefore, we define the interphase region on the basis of structural heterogeneities (density) within the model nanocomposite systems. For this, we probe the density of the atom mass of the PEO chains, ρ(*r*), as a function of the radial distance from the center of mass of the SiO_2_ nanoparticle, *r*. For this, polymer configurations are analyzed as a function of the radial distances from the center of mass of the silica nanoparticle, using a binning of 0.6 Å. The density profile of the PEO chains is calculated as an average on all PEO/SiO_2_ interphases (here 8), within a given configuration, and over all configurations. Data for ρ(*r*) for different temperatures are shown in Figure 2a. It is clear that the density of the PEO chains exhibits (on average) a maximum at distances of around 4–5 Å, followed by a minimum at distances of around 7 Å from the surface of the silica nanoparticle (around 2.5 nm from its center of mass). The maximum in the density profile is due to the attractive, dispersive (van der Waals), and Coulombic PEO/SiO_2_, polymer/nanoparticle interaction. Then, a second, smaller, maximum at the PEO density is observed at distances around 9–10 Å from the SiO_2_ outer surface. As expected, the density reduces as temperature increases; nevertheless, the polymer density in the region of the first maximum and minimum of the profile is less sensitive to temperature changes than the density at longer distances from the nanoparticle.

Based on the position of the minimum in the density profile, and considering a fairly spherical shape of the SiO_2_ nanoparticle, we define the width of the PEO/SiO_2_ interfacial region within the region of 5.5 Å from the outer surface of the nanoparticle, shown with dashed line in Figure 2a. To exclude the effect of temperature on the density profile, we present in the inset of Figure 2a, the radial density scaled with the bulk value, ρ/ρ_bulk_, for each temperature. It is clear that the position of the first peak remains relatively constant with temperature, whereas the second peak shifts closer to the outer surface of the nanoparticle at lower temperatures; the latter is expected due to the increase of density as temperature decreases. Overall, the density of the interfacial region, which is also denoted in the literature as a “bound layer” for systems with attractive polymer/filler interactions, seems to be less sensitive with a decrease of temperature compared to that of the matrix region. Last, the above definition of the interphase region, which is on the order of one molecular layer, is used for the subsequent analysis of all model PEO/SiO_2_ systems.

Interestingly, despite the peak in the polymer density profile observed in the interphase region in Figure 2a, the average mass polymer density of the interphase, shown in Figure 2b, is found to be lower than that of the matrix at all temperatures investigated. This is mainly due to the excluded volume interaction effects of atoms that belong to either the nanoparticle or the narrow (2D-like) adsorbed polymer layer, which creates low-density regions around the maximum peak. Moreover, as expected, a decrease in the polymer mass density, averaged over the matrix and interphase regions, is observed with increasing temperature. The mass density profile for the matrix region is close to the PEO bulk system for all temperatures investigated, as can be shown in Figure 2b.

We shall note that, not surprisingly, the average density of the interphase depends strongly on its exact definition, i.e., on the region over which averages are computed. More specifically, if the interphase is defined by the region that does not include the free volume region (between 22 and 25 Å from the center of the NP), the density at *T* = 220 K is found to be higher than that of the bulk region, 1.26 g cm^–3^. On the other hand, the density of the region close to the NP (between 18.5 and 22 Å) is found to be lower, namely, 0.35 g cm^–3^. A detailed discussion on the effective mass density of interphase and matrix regions within the PEO/SiO_2_ systems in the glassy regime (well below *T*_g_) as a function of the silica volume fraction can be found in our previous work.^81^

### 3.2 Density of the PEO/SiO_2_ Hybrids during Deformation

**Figure 3:**
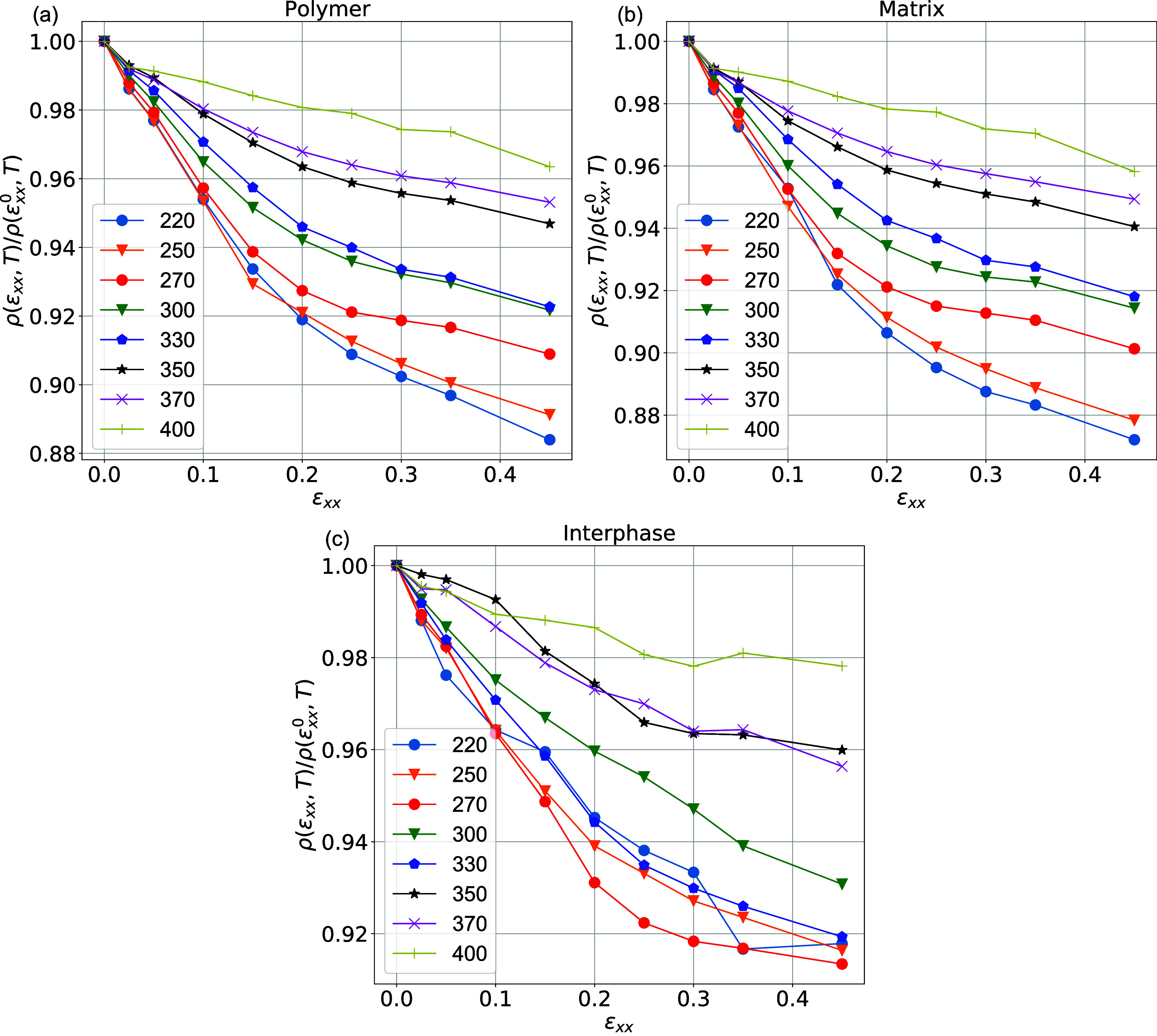
Evolution of the average density normalized over the density at equilibrium ρ(ϵ_*xx*_,*T*)/ρ(ϵ_*xx*_^0^,*T*) of (a) polymer, (b) matrix, and (c) interphase regions as a function of the deformation for PEO/SiO_2_ systems at temperatures ranging from 220 K up to 400 K. The polymer region contains both the interphase and the matrix ones.

Next, we investigate the density and structure of polymer chains, focusing on the PEO/SiO_2_ interphases, as a function of the deformation. To this end, Figure 3 presents the evolution of the average density of the entire polymer matrix, as well as the interphase (see Figure 2) and the matrix regions normalized over the density of each region at equilibrium. Note that the term “polymer region” includes both the “interphase” and the “matrix” regions, whereas the bulk term concerns the homogeneous PEO system. Density data are presented as a function of deformation; data at ϵ_*xx*_ = 0 correspond to PEO/SiO_2_ systems at equilibrium.

An interesting first observation based on the data shown in Figure 3 is that during deformation, the average density of the polymer chains and the overall hybrid material, at a given temperature, decreases. This density decrease is much more pronounced at temperatures below *T*_g_ (*T* = 220 and 250 K) but also occurs in the higher temperature range. In other words, as discussed below, the Poisson’s ratio is below 0.5, even at temperatures well above *T*_g_. For example, when the strain increases from 0.0 to 0.45, the effective density of the polymer chains drops from 1 to 0.92 at 330 K and from 1 to 0.96 at 400 K. At the same time, the effective density of the interphase drops from 1 to 0.92 at 330 K and from 1 to 0.98 at 400 K. The same behavior is also observed for the matrix region at all temperatures examined. We should note that the density of the SiO_2_ nanoparticles during the deformation remains, as expected, almost constant. The above findings indicate a potential drawback for computational works in the literature involving nonequilibrium (deformation) simulations under constant volume, i.e., assuming a Poisson’s ratio of 0.5.

The decreasing of the effective mass density of the polymer in Figure 3 is naturally related to the increase in the corresponding volume of the simulation box; the latter can be observed by examining the size of its edges during deformation (evolution of *L*_*x*_, *L*_*y*_, and *L*_*z*_). The decrease rate in the polymer mass density for *T* = 220 K is much higher than for *T* = 400 K. As expected, such changes directly correlate with the evolution of Poisson’s ratio (the ratio between the lateral to the longitudinal deformation); as the temperature increases, the Poisson’s ratio increases (more details will be provided in the next section). Lower Poisson’s ratio indicates the smallest contraction in the lateral direction *L*_*y*_ and *L*_*z*_, leading to an increase in volume during deformation.

**Figure 4:**
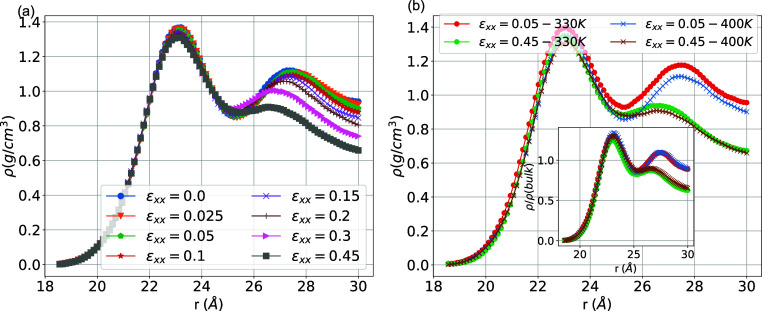
(a) Mass density profile of PEO as a function of the radial distance from the center of the SiO_2_ nanoparticle for different strain values (*T* = 330 K) and (b) comparison between the mass density profile (inset: normalized density profile over the bulk values) for two different strains (ϵ_*xx*_ = 0.05 and ϵ_*xx*_ = 0.45) and two temperatures (*T* = 330 K and *T* = 400 K).

Next, we investigate the mass density of the polymer as a function of the distance from the SiO_2_ nanoparticles and for a given strain ρ(*r*,ϵ_*xx*_); data for ρ(*r*,ϵ_*xx*_) are presented in Figure 4 (system at *T* = 330 K). Note that the data for ρ(*r*,ϵ_*xx*_) are shown up to distances that correspond to about half the distance between the nearest nanoparticle. The first peak in the density profile shown in Figure 4a,b corresponds to atoms belonging to the interphase. As the strain increases, the density profiles keep this first peak more constant for the strains corresponding to elastic behavior. The decrease of the second peak in the effective mass density of the polymer upon increasing the deformation is naturally related to the increase of the corresponding volume of the simulation box; this can be observed by probing the size of its edges during deformation. A slight decrease in the first peak (interphase region) is observed.

Figure 4b presents the density profiles for the model as a function of temperature and for two specific strains, one in the linear elastic region (ϵ_*xx*_ = 0.05) and another in the plastic region (ϵ_*xx*_ = 0.45). Regimes of high and low monomeric mass densities are observed at small distances (around 5 Å from the outer surface of the silica nanoparticle) similar to the equilibrium profile discussed above. Interestingly enough, the first density peak is independent of the strain and temperature for the range of the examined strains and temperatures.

Similarly to the data shown in Figure 2, an interfacial regime is defined for distances of up to 5–6 Å, from the outer surface of the SiO_2_ nanoparticle, for the system under investigation. At longer distances, bulk density is attained. The mass density is mainly affected by deformation, and a slight influence of temperature is observed in Figure 4b. Data for both temperature (330 and 400 K) at equilibrium (ϵ_*xx*_ = 0) are presented in Figure 2. We attribute this change to the dilation of the box volume. Note that the second peak in the density profile of PEO chains, which is observed at distances of about 10 Å, gradually decreases with temperature and during deformation. Normalization of the density over the bulk values (inset of Figure 2b) shows the independence of the density profile on temperature (excluding the effect of the temperature), and the deformation effect remains unchangeable.

Overall, the data shown in Figures 3 and 4 demonstrate that due to the rather strong density heterogeneity’s in the specific PEO/SiO_2_ nanocomposites, it is necessary to describe separately the interfacial behavior in the nanocomposite mechanical response under external deformation. More specifically, in any three-phase micromechanical model, the elastic properties of the matrix and the interphase region should depend on the temperature under consideration.^81^

## 4 Overall Mechanical Properties of the PEO/SiO_2_ Nanocomposites

**Figure 5:**
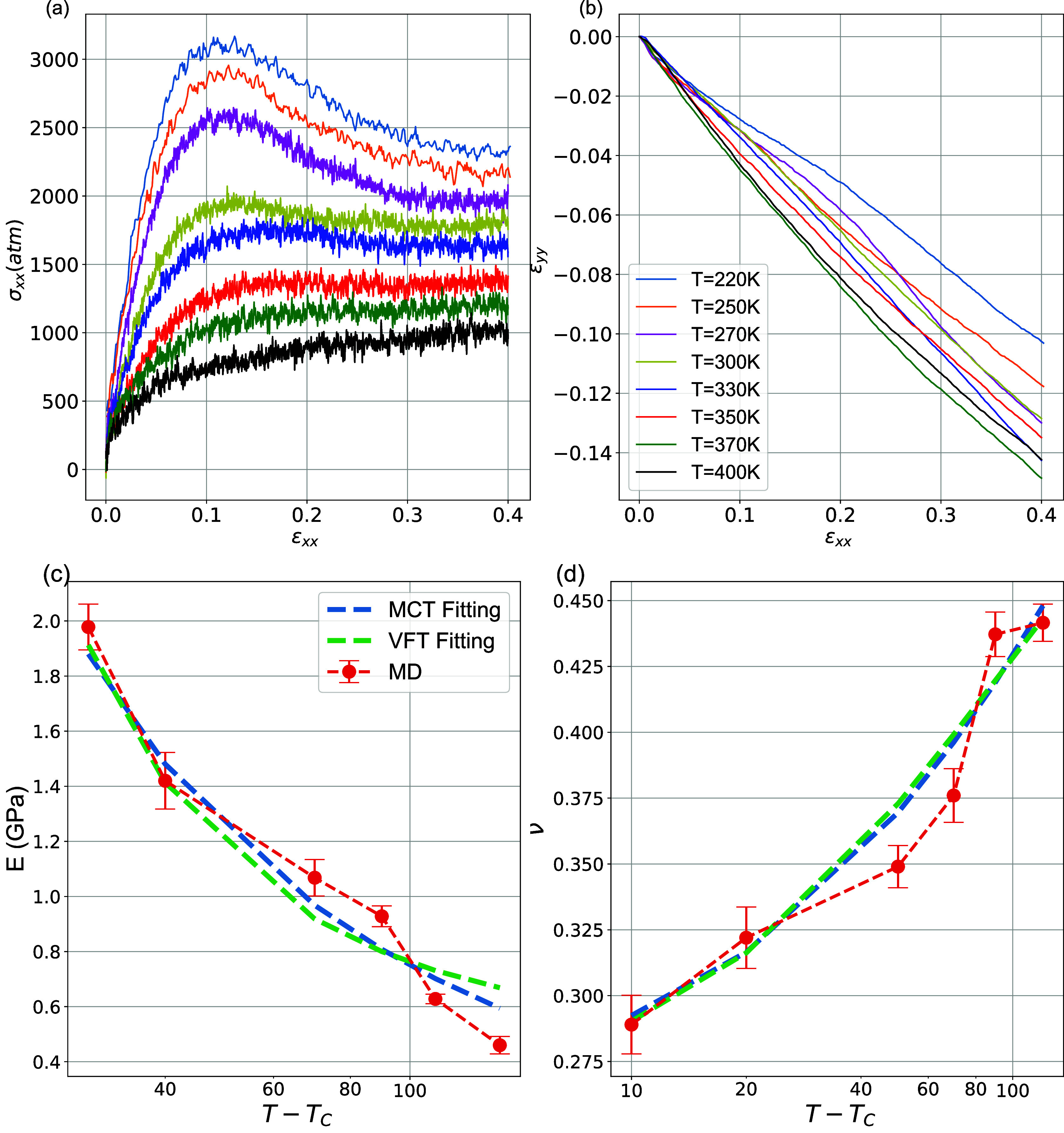
Average (global) stress–strain curves for PEO/SiO_2_ nanocomposites for different temperatures. (b) Lateral deformation in the *y* direction as a function of the applied tensile strain in the *x* direction. (c,d) Temperature dependence of the average elastic Young’s modulus and Poisson’s ratio computed for strain values up to 0.1, respectively, as a function of temperature.

We continue to analyze the mechanical response of the model PEO/SiO_2_ systems under deformation by investigating the temperature dependence of their mechanical properties. The simulation results for the effective elastic properties, i.e., Young’s modulus *E* and Poisson’s ratio ν, are extracted from the stress–strain and longitudinal–transverse strain data, within the low-strain regime for strain values up to about 0.1. Data about *E* and ν are shown in Figure 5. The strain is calculated as the global engineering one (average). As the temperature increases from 220 to 400 K, the material becomes, as expected, softer, as demonstrated in Figure 5a,b. As shown in Figure 5a,c, the elastic Young’s modulus decreases monotonically with temperature, from 3.5 GPa at 220 K to 0.48 GPa at 400 K, while Poisson’s ratio increases, from a value of approximately 0.3 at 220 K to 0.45 at 400 K. The above behavior is in good agreement with experimental results for the PS/SiO_2_ nanocomposite, where a drop in Young’s modulus from 2.5 GPa at 293 K to 0.45 at 358 K was reported.^34^ In addition, as the temperature increases from 220 to 400 K, the yield strain shows a downward trend, while the yield stress shows an upward trend. The above data are in qualitative agreement with experimental data for carbon fiber-reinforced vinyl ester polymer^37^ and predictions of micromechanical models for PS and PMMA with silica nanoparticles.^52^ We shall note that similar PEO/SiO_2_ systems at a lower temperature (150 K) exhibit a Young’s modulus of about 4.2 GPa.^65^

The above changes in the mechanical behavior of the nanocomposites as the temperature increases (that is, the reduction of strength and the appearance of a rather broad plastic-like region) are consistent with the one observed when decreasing the strain rate.^65^ The analysis of the results presented in Figure 5 shows that both the peak of the yield and the strain yield vary as the temperature increases. The yield strain decreases with temperature, implying that the material becomes softer, whereas the yield strain increases as the temperature rises. In the case of yield stress dependence, this behavior has also been reported in coarse-grained homopolymer simulations using generic bead spring models.^68^

From a theoretical point of view, the temperature dependence of the mechanical properties of polymeric systems is usually described via semiempirical relations or MCT. The latter predicts a temperature dependence for the elastic modulus and the Poisson’s ratio in the melt region via a power law divergence defined as
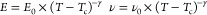
1where *T*_c_ is the transition temperature of the nanocomposite and γ is considered as the activation energy needed to activate the process of relaxation and the breakage of secondary bond energy (van der Waals interaction).^48^

Besides the above, the Vogel–Fulcher–Tammann (VFT) equation was also proposed to describe the temperature dependence of the Young’s modulus and the Poisson’s ratio, as
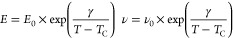
2where *T*_C_ is the temperature at which the system viscosity diverges and β is the activation energy.^68^ We should note here that both the MCT and VFT models are expected to be more accurate for describing the behavior of polymeric systems above the *T*_g_.

In Figure 5c, the Young’s modulus is plotted as a function of *T*–*T*_c_, where the divergence of the power law should appear in an exponential form, and the critical temperature is obtained by fitting the data using the law mentioned above. The fitting parameters for both Young’s modulus and Poisson’s ratio are presented in Table 1. It is clear that the values of Young’s modulus and Poisson’s ratio closely follow the dependence of VFT and MCT over the examined temperature range, thus implying that the principle of time–temperature superposition holds on the short time scale of the elastic response. For the modeled PNC, the critical temperature extracted from Young’s modulus is found to be slightly lower than the glass-transition temperature, *T*_g_ = 280 K, and also lower than the critical temperature of pure PEO (*T*_g_ = 270 K). However, *T*_C_ is found to be 220 K from the VFT theory extracted from the Poisson’s ratio, as we can see from Table 1.

**Table 1:** Fitting Parameters from MCT and VFT Models

	*E* (GPa)			ν		
	*E*_0_ (GPa·K)	*T*_c_ (K)	γ	ν_0_ (K)	*T*_c_ (K)	γ
MCT	14	267	–0.65	0.11	259	0.28
VFT	0.47	259	51	0.58	220	–50

An important phenomenon in polymeric nanocomposites containing inorganic fillers is their nonlinear dynamic viscoelastic behavior for *T* > *T*_g_; for nanofiller’s filled rubbers, the latter is usually described as the Payne effect or the Mullins effect.^68−72,104^ To investigate such a nonlinear behavior, the elastic modulus of the PEO/SiO_2_ systems is further computed as a function of strain. To obtain a smooth elastic modulus–strain curve, we first fit the stress–strain curve with the following expression derived from the well-known nonlinear stress–strain relations of polymer elasticity^1,105−107^
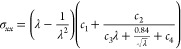
3where all *c*_*i*_ are fitting parameters, σ_*xx*_ is the tensile stress, and λ – 1 is the tensile strain, ε_*xx*_. The Young’s modulus was computed subsequently via the derivative of stress with respect to the strain. The above equation is expected to be valid for systems at *T* ≥ *T*_g_, as it is inapplicable for low temperatures due to the existence of strain hardening and softening regimes.

**Figure 6:**
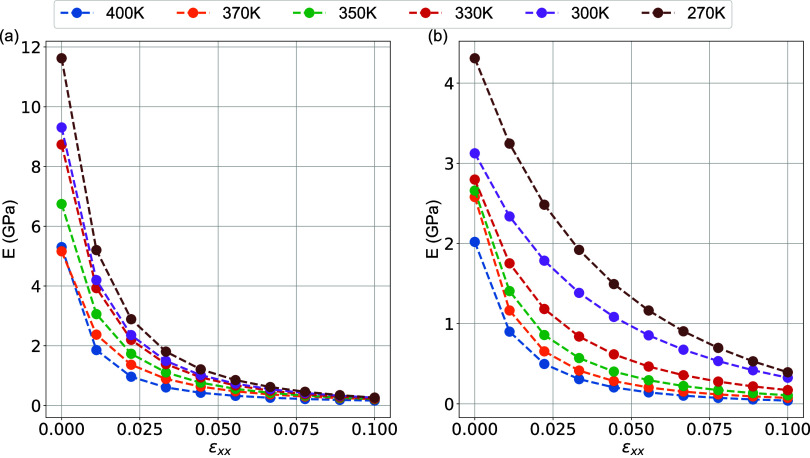
Elastic modulus of PNCs as a function of strain for different temperatures: (a) PEO/SiO_2_ hybrids and (b) bulk PEO systems.

The results on the dependence of the elastic modulus on the strain rate are shown in Figure 6. As expected, the elastic modulus drops dramatically with increased strain, especially at small deformations to reach a plateau in the case of the PNC system, which is analogous to the change of dynamic storage modulus with the shear amplitude reported in previous works.^68,69,104^ Such strain-induced nonlinear behavior of elastic modulus can serve as an indirect indicator of the Payne effect. As the temperature increases, the elastic modulus reaches the plateau faster, indicating a less distinct nonlinearity of elastic modulus with the strains, as well as mirroring high prominent nonlinear dynamic viscoelasticity. The Payne effect for bulk PEO systems is weaker compared to the PEO/SiO_2_ hybrids for all temperatures studied here.

## 5 Distribution of Mechanical Properties in PEO/SiO_2_ Nanocomposites

In this part, we examine the spatial distribution of mechanical properties in heterogeneous PEO/SiO_2_ systems, as defined at the “local” level, by independently analyzing the polymer chains at the polymer/nanoparticle interphase and in the matrix region. To investigate the spatial distribution of the mechanical properties of the model PNC systems, in addition to the global stress and strain calculations discussed above, the stress and strain are calculated at the atomic level resolution as discussed in Section 2.^97^

**Figure 7:**
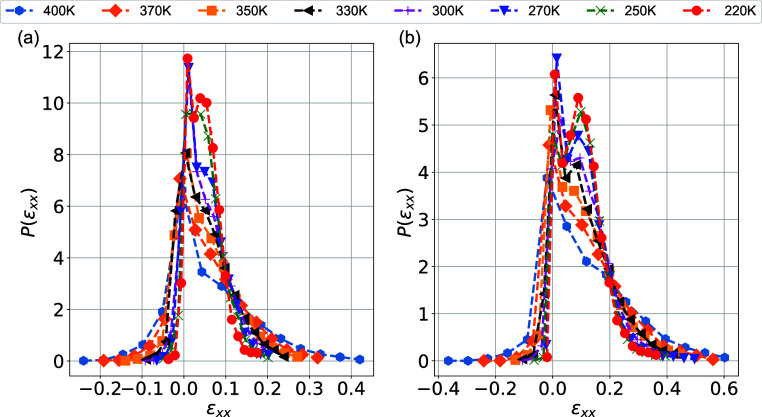
Probability density function of the local strain for the overall PEO/SiO_2_ hybrids at different temperatures for (a) ϵ_*xx*_ = 0.05 and (b) ϵ_*xx*_ = 0.1 (elastic region).

To further investigate and visualize the typical error related to the heterogeneous distribution of local strain fields for the model PEO/SiO_2_ under deformation as a function of temperature, we perform a 3D domain decomposition in the simulation domain, into small cubic boxes of length 5 Å, and compute the average strain, within each box, for a given applied global deformation ϵ_*xx*_ = 0.05 and ϵ_*xx*_ = 0.1 (values in the linear-like region). Data about the probability density function of the strain, *P*(ϵ_*xx*_), are shown in Figure 7. As expected, in the linear regime, symmetric distributions are shown with values around the globally applied one. As we can observe from the data shown in Figure 7, *P*(ϵ_*xx*_) exhibits a clear peak exactly in the global value (0.05 or 0.1). A second small peak appears close to 0 corresponding to the strain in the rigid silica nanoparticle regions where, due to the higher stiffness, the deformation is almost negligible. The narrow peaks in the distributions shown in Figure 7 indicate that most atoms reproduce the applied global strain, while the broader distribution at high temperatures indicates a more heterogeneous strain field within the model PNCs.

**Figure 8:**
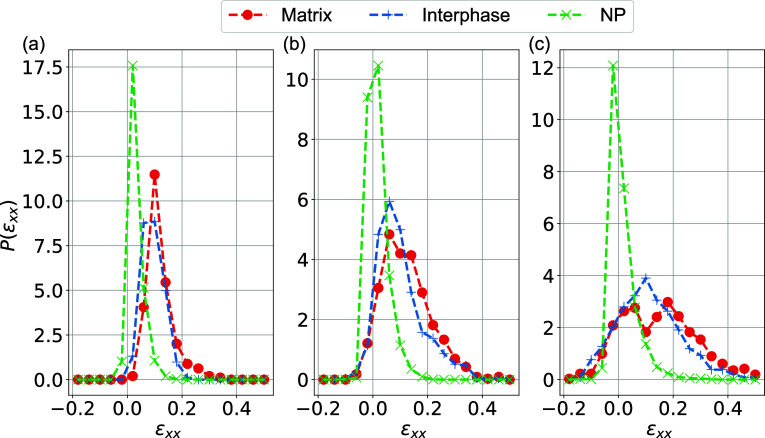
Probability density function of local strain, *P*(ϵ_*xx*_), in three different regions (matrix, interphase, and silica nanoparticle) at (a) *T* = 220 K, (b) *T* = 330 K, and (c) *T* = 400 K.

To examine in more depth the local strain values in subdomains for a given global applied strain, ϵ_*xx*_ = 0.1, we present in Figure 8 the probability density function of strain, *P*(ε_*xx*_), for the nanoparticle, interphase, and matrix regions. First, as expected, *P*(ε_*xx*_) for the nanoparticle region (SiO_2_ atoms) exhibits a clear peak around zero due to its higher rigidity for the three investigated temperatures. In the glassy state (*T* = 220 K), symmetric distributions with values around the globally applied one are shown for the matrix region. In the interphase region, *P*(ε_*xx*_) is much broader, reflecting the high heterogeneity of the strain in this region. The broad *P*(ε_*xx*_) distribution in the interphase region reflects the strong variations and fluctuations as well of the local strain around the global one due to the increase of the thermal fluctuation effect. As the temperature increases (*T* = 330 K and *T* = 400 K), *P*(ε_*xx*_) for both regions (interphase and matrix) exhibits a wide distribution around the global applied strain. The distribution of strain in the matrix and interphase regions is close at *T* = 400 K.

**Figure 9:**
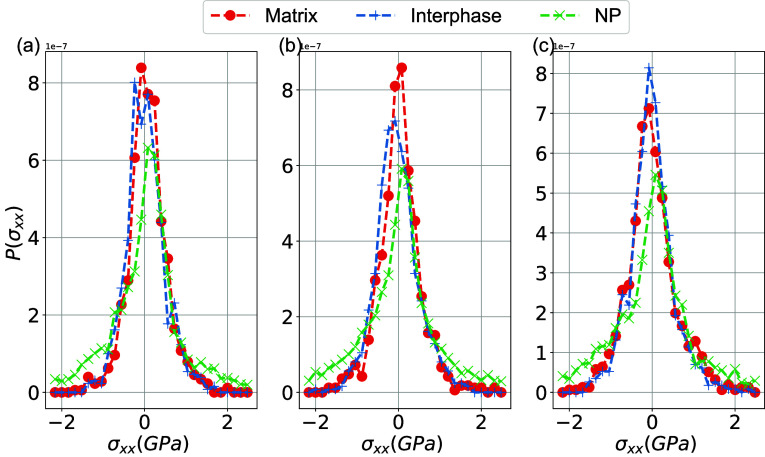
Probability distribution of the local average stress in three different regions (NP, interphase and matrix for a global strain ϵ_*xx*_ = 0.1) at (a) *T* = 220 K, (b) *T* = 330 K, and (c) *T* = 400 K.

The distribution of local stress is also quite broad for all regions, as shown in Figure 9. We attribute this strong variation of *P*(σ_*xx*_) to the part of the nonbonded interaction of the atomic Virial expression of stress (partly related to Lennard-Jones interactions). Virial stress depends on the attraction forces between the atoms, taking into account the average within a distance of *r*_cut_ which is very sensitive to distance *r*. The stress probability distribution function, *P*(σ_*xx*_), after the decomposition of the simulation domain into small cubic boxes of length 5 Å qualitatively and quantitatively follows the same behavior of the local stress distribution in the different subregions.

**Figure 10:**
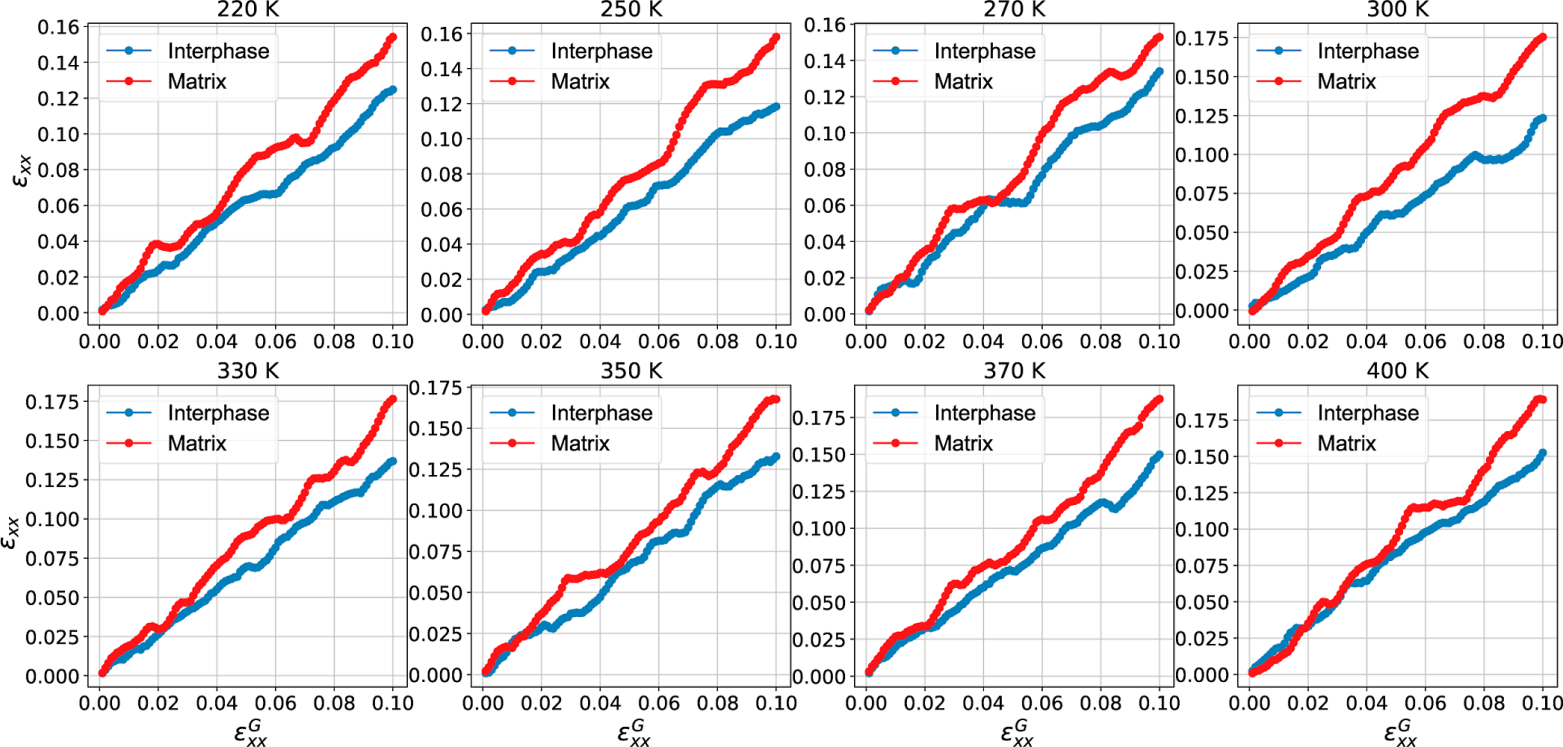
Local strain field in the interphase and matrix region at different temperatures as a function of the global applied engineering strain, focusing in the liner regime (up to 0.1 strain).

Figure 10 shows the average local strain in the interfacial and matrix regions, as a function of the global strain applied (deformation steps), focusing on the linear regime, for the PEO/SiO_2_ systems at different temperatures. The data are obtained by averaging the local strain data within each region. In particular, during the deformation process, an almost affine strain field is produced in the bulk (homogeneous) sample (data not shown here), but, in PNCs, the presence of highly stiff nanofillers leads to a nonaffine strain field in the sample.^65^ Thus, for the strain values studied here, nanoparticles practically do not experience any strain, but their presence alters the local strain within the polymer chains located in the vicinity of the nanoparticle. We should mention that a rather broad distribution of strain field within the interphase region is observed, due to different responses of different parts of it which cannot be captured in the average data.^89,97,108^ As shown in Figure 10, the average value of local deformation in the far-field matrix region of the PEO/SiO_2_ nanocomposites is slightly higher than the corresponding value at the interphase, mainly at lower temperatures. These findings indicate that the interphase is less deformed and exhibits a reduced mobility during deformation compared to the matrix, for all but very high temperatures. When the temperature is increased, the deviation between the strain in the matrix and the interphase region decreases. The increase in local deformation within the interphase and the matrix region reflects the softening of the material during heating.

**Figure 11:**
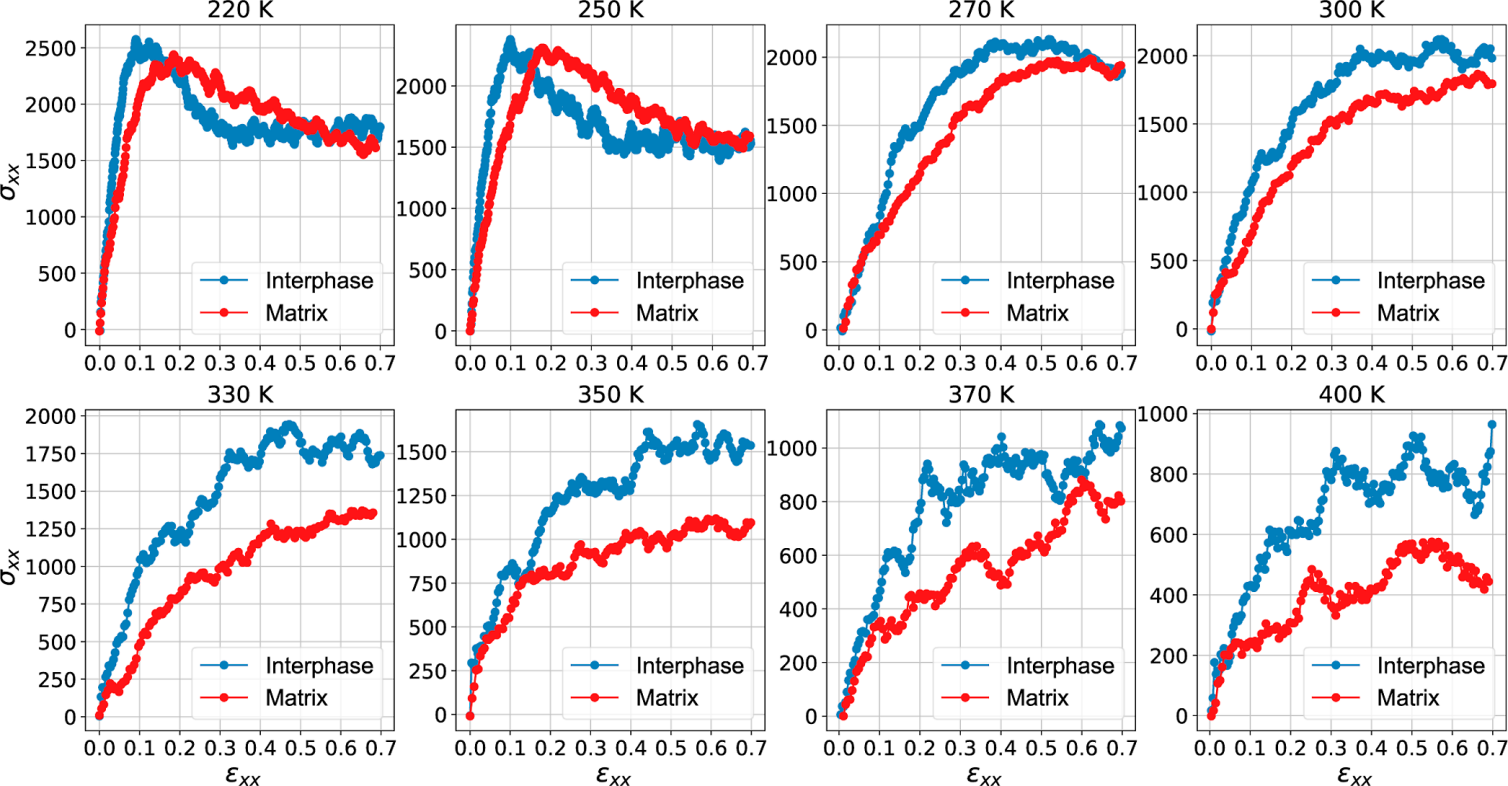
Local stress–strain field at the interphase and matrix regions at different temperatures.

Next, we directly examine the spatial distribution of the mechanical properties of the PO/SiO_2_ hybrids by computing the stress and strain fields in the two investigated regions. Figure 11 displays local stress versus local strain data, which can be used to calculate the engineering constant for each region as a function of temperature. As expected, the stress–strain behavior of both the matrix and interphase PEO regions tends to decrease as the temperature increases. Interestingly enough, the polymer/silica interphase region is more rigid than the primary matrix because of the high stresses. A clear linear elastic region (ϵ_*xx*_ = 0.1) is observed for the interphase and matrix regions when the temperature is below *T*_g_ (220 and 250 K). This linear region starts to disappear as the temperature increases, reflecting the increase of the nonlinear stress–strain dependence in the melt state. This is not surprising, as the viscoelastic behavior of the polymer matrix dominates that of polymer chains at high temperatures.

**Figure 12:**
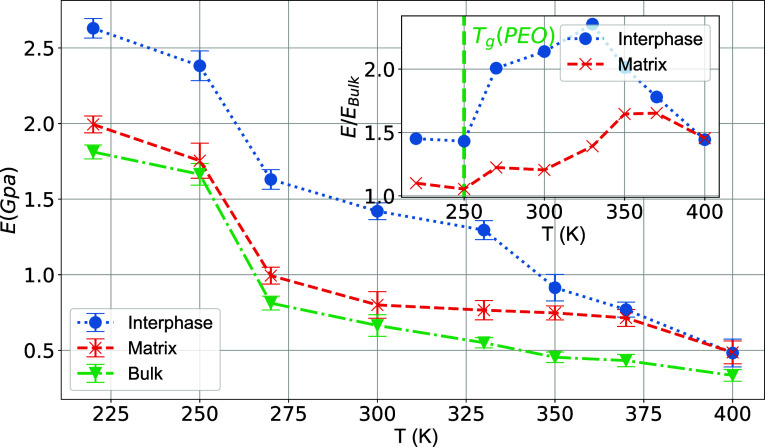
Temperature dependence of the Young’s modulus for the interphase and matrix regions in PEO/SiO_2_ nanocomposites. Data about the bulk PEO systems are also shown. Error bars are computed over several (here five) uncorrelated configurations. The inset shows *E*(*T*)/*E*_bulk_(*T*) for interphase and matrix regions. The dashed line in the inset denotes the *T*_g_ of the bulk model PEO chains.

Young’s modulus for each region is calculated through the slope of the stress–strain data in the low-strain, linear like regime (up to 0.1 strain values), where the stress is linear depending on the strain; results are shown in Figure 12. In the inset of Figure 12, we present the variation of Young’s modulus for both regions normalized over Young’s modulus for the bulk (homogeneous) PEO system. For reference, the Young’s modulus of the matrix region drops from 1.9 GPa at 220 K to 0.51 at 400 K. The interphase region shows a similar drop from 2.62 GPa at 220 K to 0.56 GPa at 400 K. The interphase and matrix regions show an increase in Young’s modulus with respect to the bulk value when T is higher than *T*_g_ of the pure bulk PEO system (around 150 K), while it remains constant at *T* < *T*_g_. The normalized Young’s modulus for the interphase region shows an increase of up to *T* = 330 K, while in the matrix region, it increases to *T* = 370 K and then drops (inset of Figure 12). These findings indicate that the macroscopic properties of the PNC depend strongly not only on the bulk and nanoparticle properties of their constitutive components but also on the nature of the polymer/fill interactions and the behavior of the matrix region where the rigidity can be greater up to 1.5 times than the normal bulk material at a specific . We should note that the rigidity of the PEO/SiO_2_ interphase, deep in the glassy state (e.g., *T* ≃ 150 K well below *T*_g_), is about 2.5 times higher than that of the PEO matrix.^81,97^

Using the MCT model (via eq 1) for the different regions, the critical temperature extracted from Young’s modulus is found to be around 269 K for all regions, close to the one found if we consider the average data over the entire PEO/SiO_2_ system. The value of the critical exponent, γ, is approximately equal to 0.12 in the interphase, 0.08 in the matrix, and 0.115 in the bulk region. As expected, the activation energy for the interphase region is much higher than that for the matrix region, indicating the high rigidity of the interphase compared to that of the matrix region. However, contrary to our expectations, the bulk region presents an activation energy higher than that of the matrix in the nanocomposite, probably due to the high heterogeneity of local mechanical behavior in the pure polymer region.^81^

**Figure 13:**
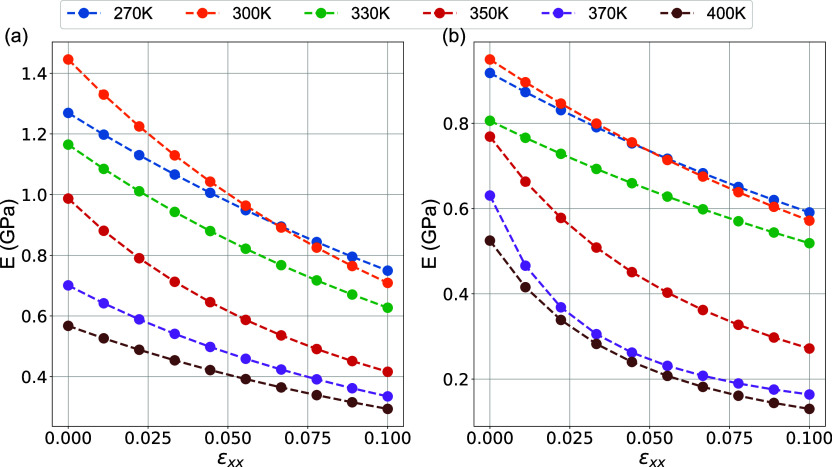
Elastic modulus of PEO/SiO_2_ hybrids as a function of strain for different temperatures. (a) Interphase and (b) matrix regions.

In Figure 13, the dependence of the elastic modulus on the tensile strain for the interphase and matrix regions is plotted for low strain values up to about 0.1. Data are obtained by fitting using equation eq 3. The elastic modulus for the interphase drops slowly when the system is subjected to deformations at different temperatures but drops more abruptly in the matrix region with a further increase in the temperature and reaches an asymptotic value at relatively large strains. Consequently, the viscoelastic behavior of the interphase region is different from that of the matrix and deserves further investigation. In particular, the initial elastic modulus, which gives the indication of mechanical reinforcement, is seen to be higher for the interphase region. Furthermore, the asymptotic elastic modulus is also seen to be higher in the interphase region compared to the matrix region. Interestingly enough, the Payne effect for local regions (interphase and matrix) is less dominant compared to nanocomposite and bulk systems (Figure 6) and for all temperatures under investigation where the elastic modulus decays slower and does not reach the plateau at higher systems.

## 6 Mobility of Polymer Chains under Deformation

In the last part of our study, we investigate the correlation between the mechanical response of PEO/SiO_2_ systems and their mobility by directly probing the mean-square displacement (MSD) of polymer atoms for a specific deformation (value of strain, ϵ), Δ**R**(ϵ(*t*)), defined as Δ**R**(ϵ(*t*)) = ⟨(**R**(ϵ(*t*)) – **R**(ϵ(0))^2^⟩, for different temperatures. The affine deformation *x*_0_^*m*^ϵ_*xx*_ is removed from the MSDs along direction “*x*” (deformation), while ν*y*_0_^*m*^ϵ_*xx*_ and ν*z*_0_^*m*^ϵ_*xx*_ are removed from the MSDs in “*y*” and “*z*” directions, with *x*_0_^*m*^, *y*_0_^*m*^, and *z*_0_^*m*^ being the initial positions of atom m at equilibrium and ν is the Poisson’s ratio at the actual temperature (Figure 5). In the above relation, **R**(ϵ(*t*)) and **R**(0) are the positions of the atoms at time *t* and 0, respectively, and the brackets denote a statistical average over all polymer atoms and all possible time origins. Δ**R**(ϵ(*t*)) can be decomposed into axial (*X* direction, Δ*R*_*x*_) and radial (*Y*, *Z* directions, Δ*R*_*yz*_) components and calculated for the different temperatures. Moreover, we further investigate the motion of the polymer chain in the interphase and matrix regions.

**Figure 14:**
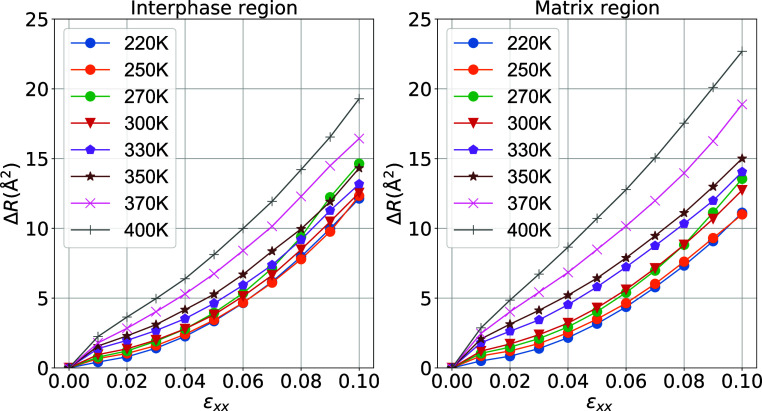
Evolution of the components of the mean-squared displacement for interphase and matrix regions as a function of the strain at different temperatures.

As presented in Figure 14, the behavior of the polymer mobility is influenced by changes in temperature during the uniaxial deformation process for the interphase and matrix regions. A nonmonotonic change occurs for different temperatures in chain motion in both regions, as observed in Figure 14. As the temperature increases, the MSD in both regions increases, reflecting the softening of the subdomains upon heating the system. The MSD in the matrix region is higher than in the interphase region, which is consistent with the local deformation results found in Figure 10. We can correlate the preceding to the fact that, due to the attractive filler, the bound (interfacial) layer is less mobile than the matrix.

**Figure 15:**
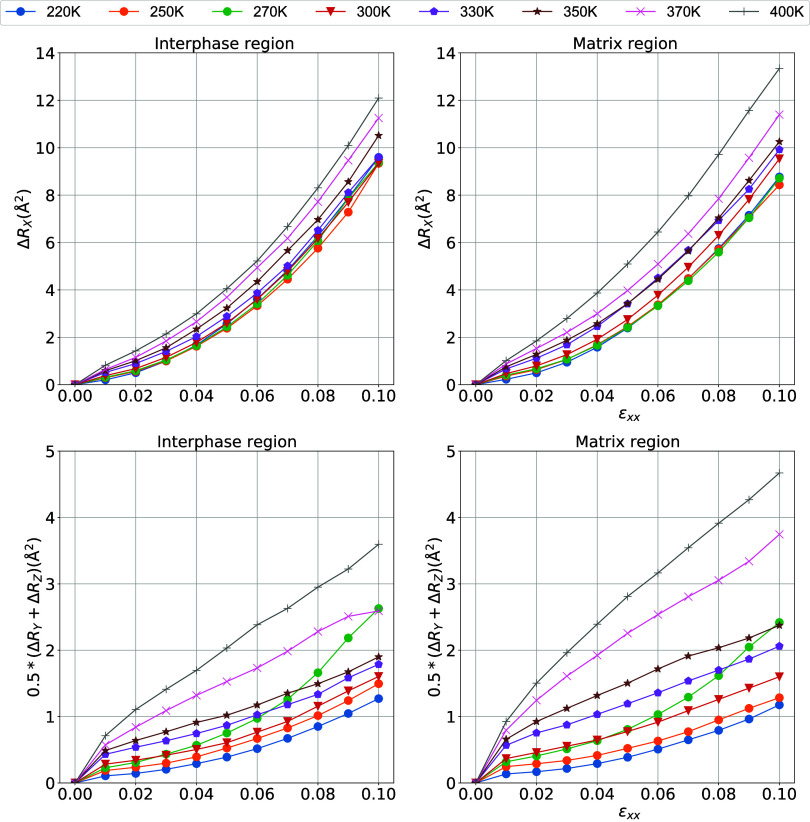
Evolution of the parallel (*x*) and average of the perpendicular (*y* and *z*) curves to the deformation components of the mean-square displacement of polymer atoms in the PEO/SiO_2_ interphase and matrix regions as a function of the strain and for different temperatures.

To further investigate the temperature dependence of the local mechanical properties, we decomposed the motion of polymer chains into axial and radial motions under different temperatures. Then, we probe the parallel (*x*) and perpendicular (averages of *y* and *z*) curves to the imposed deformation components of PEO MSDs that can serve as a reference for the evolution of the rigidity during axial deformation. To this end, in Figure 15, the axial (*x*-component) and radial (average of *y*- and *z*-components) directions of the MSD are presented. It is clear from the above data that the polymer motion behavior in the axial direction in the interphase regions has nearly no significant effect by temperature changes during the uniaxial stretching process, as indicated by the almost coincident axial MSD curves for different temperatures. However, a more pronounced effect is observed in the radial directions for both regions. In this case, the axial deformation of polymer chains in the interphase region is controlled and limited by the presence (entropic term) of the silica nanofiller and their strong attraction (enthalpic term) to it.

Furthermore, the radial MSD curves presented by the average *y* and *z* components of MSD show that the range of chain motion increases dramatically with increasing temperature from 220 to 400 K. If the two ends of the PEO chain are fixed at the NP surface (bridge or loop chains), the distance of the PEO chain motion that increases with temperature in the radial direction would result in a larger angle, which would form a peak between the chain and the axial direction. Higher mobility implies that the PNC model becomes softer at higher temperatures. These findings are in agreement with the mechanical properties found in Figure 5. With increasing temperature, the rigidity of both regions decreases, as well as the disparity between the values of the radial deformation components of the mean-square displacement of the polymer in the interphase and matrix regions. This behavior can be ascribed to changes in the conformation within the matrix region upon increasing the temperature. A detailed investigation of such effects will be the subject of a future study.

## 7 Discussion and Conclusions

Atomistic simulations can be a valuable tool to elucidate the mechanical behavior and mobility of polymer chains in nanostructured polymer materials. In the present work, we studied the (heterogeneous) mechanical behavior of nanocomposites consisting of PEO chains with silica nanoparticles, and particularly its temperature dependence, via atomistic MD simulations under tensile deformation. PEO/SiO_2_ is a model nanocomposite system of well-dispersed nanofillers due to attractive polymer/nanoparticle interactions. We probe the mechanical response of the model systems by investigating the variation of the effective mechanical properties, the evolution of the radial mass density, and the mobility of polymer chains at temperatures across a range of temperatures in the transition from the glassy toward the melt regime. To investigate spatial heterogeneities, we directly probed the response of the PEO/SiO_2_ model systems in the interphase and matrix regions by computing stress and strain fields at a local, per atom, level.^89,97^ The behavior of the model PEO/SiO_2_ systems changes dramatically as we increase the temperature to reach approximately the liquid state at 400 K where the Poison’s ratio at this temperature is close to the liquid case (around 0.45). As expected, the mechanical behavior of both the interphase and matrix regions, in the PEO/SiO_2_ systems, strongly depends on the temperature. The rigidity at the interphases is larger than that in the matrix region across the entire range of temperatures studied here; however, with increasing temperature, the rigidity at the interphase approaches that in the matrix region. Furthermore, we emphasize that the mass density is mainly affected by deformation and a slight influence of temperature is observed, (see data in Figure 4 a,b); that is, the conformations of the polymer chains remain unaffected by the temperature, which can be observed in the radial mass density profiles. To further explore the effect of temperature on the mobility of polymer chains in PEO/SiO_2_ systems, we calculated the mean-square displacement in the interphase and matrix regions. In general, the mobility of chains in the matrix region is higher than that in the interphase, and this difference increases with temperature. Furthermore, the chain motion in the axial direction in the matrix region is marginally affected by changes in temperature during the uniaxial stretching process. On the contrary, a more pronounced effect is observed in the interphase region. Consequently, the decrease of the axial mechanical properties (Young’s modulus) is due to the softening of the interphase region as the temperature rises. However, the radial motion (represented by the *Y* components of MSD) shows a profound increase in both regions; hence, we can deduce that both regions influence the global Poisson’s ratio. Finally, we should state, we expect that the behavior of the model nanocomposites will be different during plastic deformation, where the stress does not depend linearly on the applied strain. The mechanism of heterogeneity of mechanical properties is also expected to be different in the plastic region. On these grounds, the effect of polymer conformations on the mechanical reinforcement of PNCs in the plastic region at different temperatures is a subject worth detailed investigation. For example, future work could focus on capturing the deformation and mobility of polymer chains in the plastic regime, as well as their relaxation back to equilibrium after cessation of the imposed loading.

## Supporting Information Available

Force field description for the PEO/SiO_2_ model and atomistic simulation; thermodynamic equilibrium; detailed descriptions of computation of local stress–strain; dynamics of the polymer chains in the interfacial zone; determination of glass-transition temperature; and additional references (PDF)
