# Supplementary material for: Distribution of Mechanical Properties in Poly(ethylene oxide)/silica Nanocomposites via Atomistic Simulations: From the Glassy to the Liquid State
Source: Macromolecules. 2024 Apr 29;57(9):3967–84. doi: 10.1021/acs.macromol.4c00537 (PMC11190983; doi:10.1021/acs.macromol.4c00537)
Supplement: Supplementary file 1 — ma4c00537_si_001.pdf [file ma4c00537_si_001.pdf]

Supplementary Information
Distribution of Mechanical Properties in Poly(ethylene oxide) / Silica Nanocomposites via Atomistic Simulations: From the Glassy to the Liquid State

Hilal Reda ,[†] Ioannis Tanis,[†] and Vagelis Harmandaris[*,‡,¶,†]

†*Computation-based Science and Technology Research Center, The Cyprus Institute, Nicosia 2121, Cyprus*

‡*Department of Mathematics and Applied Mathematics, University of Crete, Heraklion GR-71110, Greece*

¶*Institute of Applied and Computational Mathematics, Foundation for Research and Technology - Hellas, Heraklion GR-71110, Greece*

E-mail: h.reda@cyi.ac.cy;v.harmandaris@cyi.ac.cy

S1 Force field description for the PEO/SiO_2 model and atomistic simulation

For the description of the PEO, a modified united atom (UA) TraPPE based force field was used,[1,2] and for the SiO_2 nanoparticle a full atom representation was used.[3] For the calculation of the electrostatic interactions the particle-mesh Ewald (PME) method was applied.[4] The simulations were performed in the NPT statistical ensemble, where the pressure was kept constant with the use of Parrinello Rahman barostat[5] and the temperature is applied using the Nose Hoover thermostat.[6] The LAMMPS simulation package was used for

the simulations.[7] All force field parameters for PEO and silica are provided in in Tables S1 and S2.

Table S1: Non-Bonded Interactions (i, j denote different atom types)

$$V_{LJ}(r_{ij}) = 4\epsilon_{ij}\left[\left(\frac{\sigma_{ij}}{r_{ij}}\right)^{12} - \left(\frac{\sigma_{ij}}{r_{ij}}\right)^{6}\right],\ r \leq R_C, \text{ Lennard-Jones}$$

Atom Types	mass (g/mol)	σ (nm)	ϵ (kJ/mol)	charge(electron units e.u.)
CH_2	14.027	0.395	0.3824	0.25
CH_3	15.035	0.375	0.3824	0.25
O (PEO)	15.9994	0.280	0.457296	-0.5
Si	28.086	0.392	2.5104	1.02
O (Silica)	15.9994	0.3154	0.636	-0.51
H	1.008	0.2352	0.092	0.255
CH_3-CH_2	–	0.385	0.558247	–
O-CH_2	–	0.3375	0.418210	–
O-CH_3	–	0.3275	0.610424	–

Table S2: Bonded Interactions

Bond	$V_b(r_{ij}) = \frac{1}{2}K^b_{ij}(r_{ij} - b_{ij})^2$					
	b_{ij} (nm)			K^b_{ij} (kJ mol^{-1} nm^{-2})		
CH_2 - CH_2	0.154			217700		
CH_2 - O	0.141			267900		
CH_3 - O	0.141			267900		
Si - O	0.163			323984		
H - O	0.095			533549		
Angle	$V_b(r_{ijk}) = \frac{1}{2}K^\theta_{ijk}(\theta_{ijk} - \theta^0_{ijk})^2$					
	θ^0_{ijk} (°)			K^θ_{ijk} (kJ mol^{-1} rad^{-2})		
CH_2 - CH_2 - O	112			418.218		
CH_2 - O - CH_2	112			502.194		
CH_3 - O - CH_2	112			502.194		
Si - O - Si	144			209.6		
O - Si - O	109.47			469.72		
Si - O - H	119.52			228.84		
Dihedral	$V_{rb}(\phi_{ijkl}) = \sum_{n=0}^{5} C^n_{ijkl}(cos(\psi))^n$					
	C^0_{ijkl} (kJ/mol)	C^1_{ijkl} (kJ/mol)	C^2_{ijkl} (kJ/mol)	C^3_{ijkl} (kJ/mol)	C^4_{ijkl} (kJ/mol)	C^5_{ijkl} (kJ/mol)
O - CH_2 - CH_2 - O	2.22267	17.03651	8.29835	-31.2451	5.13025	-1.91522
CH_2 - CH_2 - O - CH_2	1.60941	19.79231	-7.82474	-15.72474	6.43215	-4.5435
CH_2 - CH_2 - O - CH_3	1.60941	19.79231	-7.82474	-15.72474	6.43215	-4.5435

To avoid ”system size effects” in our simulations, i.e., the overall mechanical behavior and the properties computed (e.g. density profile, mechanical properties, chain dimensions, etc.) are the same if larger model systems, with the same loading of nanofillers, are used. To avoid such effects we considered large systems involving up to 8 nanoparticles. In order to improve statistics, all model atomistic PEO/SiO2 systems considered in the present work include 8 SiO2 nanoparticles and 384 PEO chains (each chain contains 150 atoms), whereas the size of the simulation cubic box at equilibrium (for one cell contains one NP) , in each direction, varies from 5.7nm at 220 K to 5.9 nm at 400 K. The counter length is 230 Å, Kuhn length is 10 Å, persistence length is 9.3 Å,[8] and radius of gyration in function of temperature are

given in table S3.

Table S3: Evolution of R_g in function of temperature

T(K)	220	250	270	300	330	350	370	400
R_g Å	13.2	14.4	15.1	16.1	16.7	17.1	17.3	17.5

S2 Thermodynamic equilibrium

To verify that the thermodynamic equilibrium is established, we monitored the potential energy, the density as well as the polymer radius of gyration. Figure S1 displays the corresponding graphs for a simulation run of 50 ns at a temperature T = 370K. Moreover, we perform additional short MD runs under NPT condition for few ns for thermal and local structure equilibration. To clarify this issue we present in Figure S2 the evolution of the energy (potential and kinetic energies) as well as the pressure during the relaxation for the replicated system at 220 K. The average values of the pressure are close to the atmospheric values. Large fluctuations in pressure are due to the incompressibility of the system and it is a common issue in MD simulations.

S3 Computation of local stress-strain

To investigate the heterogeneous mechanical behavior of the model PEO/SiO_2 systems we need to probe stress and strain field in local (atomic) level. For this, we use a per-atom calculation of stress and strain, under an imposed global strain. Local (per-atom) stress can

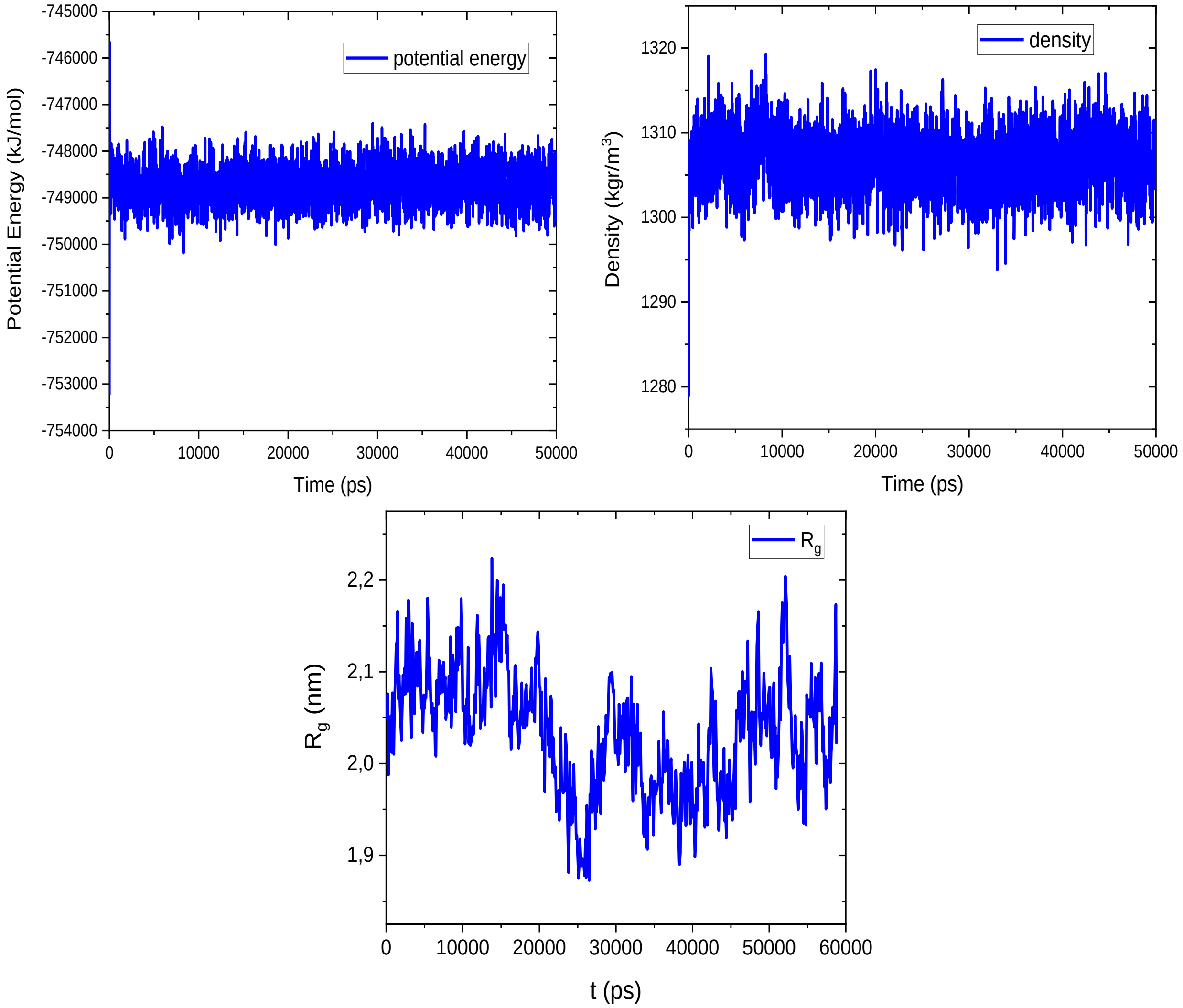

Figure S1: Time evolution of the potential energy (left), density (right) and radius of gyration (bottom) of the system T= 370 K.

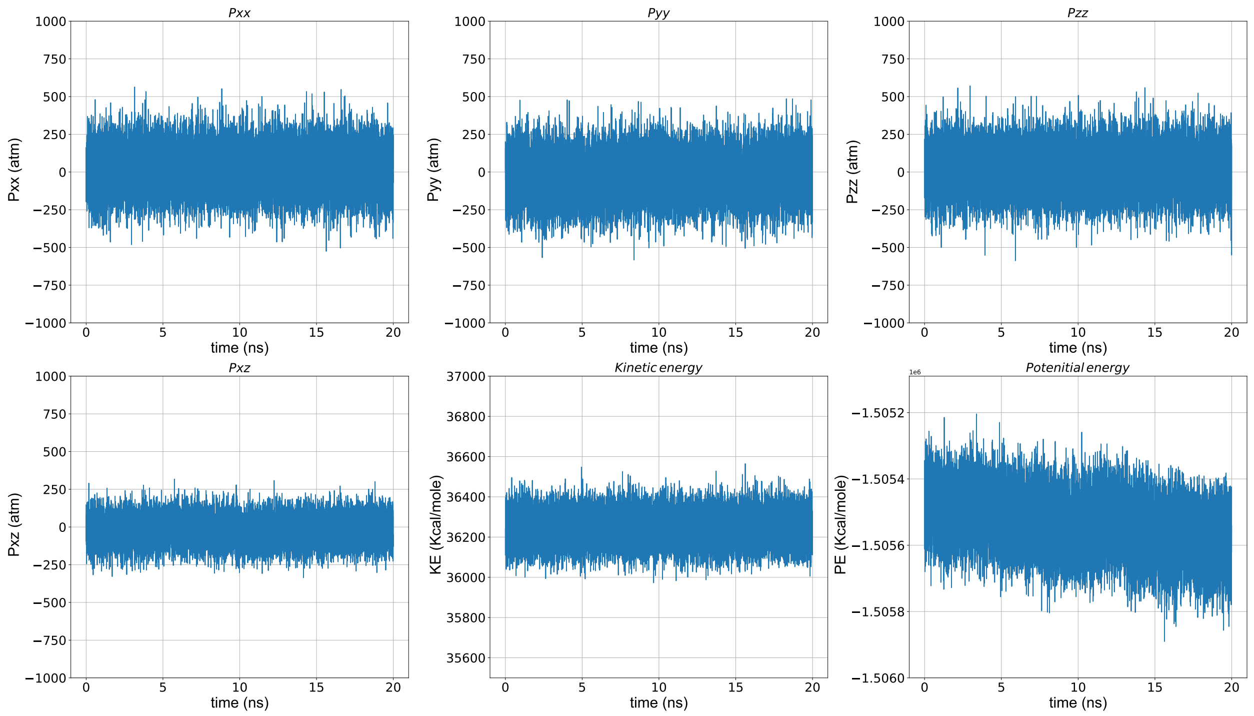

Figure S2: Evolution of pressures as well as the potential and kinetic energies during relaxation at 220 K.

be directly computed, for each atom i, σ_i, via the atomic Virial formalism through:

$$\begin{aligned}\sigma_{i_{\alpha\beta}} = -\frac{1}{V_i}\Bigg[& m v_{i_\alpha} v_{i_\beta} + \frac{1}{2}\sum_{pairs}\left(r_{i_\alpha}F_{pair,i_\beta} + r_{j_\alpha}F_{pair,j_\beta}\right) + \frac{1}{2}\sum_{bonds}\left(r_{i_\alpha}F_{bond,i_\beta} + r_{j_\alpha}F_{bond,j_\beta}\right) \\ & + \frac{1}{3}\sum_{angles}\left(r_{i_\alpha}F_{angle,i_\beta} + r_{j_\alpha}F_{angle,j_\beta} + r_{k_\alpha}F_{angle,k_\beta}\right) \\ & + \frac{1}{4}\sum_{dihedrals}\left(r_{i_\alpha}F_{dihedral,i_\beta} + r_{j_\alpha}F_{dihedral,j_\beta} + r_{k_\alpha}F_{dihedral,k_\beta} + r_{l_\alpha}F_{dihedral,l_\beta}\right) \\ & + \mathbf{K}space\left(r_{i_\alpha}F_{i_\beta}\right) + \sum_{n=1}^{N_f} r_{i_\alpha}F_{i_\beta}\Bigg], \end{aligned} \tag{S1}$$

where the indices i, j, k and l denote atoms and α and β the Cartesian coordinate system (x, y, z). Eq. (S1) contains contributions from pairwise non-bonded interactions, bond stretching, bond angle bending, and dihedral torsional potentials, a $\mathbf{K}$ space contribution from long-range Columbic interactions and finally there is a term for the N_f fixes that apply internal constraint forces to atom i. V_i is the atomic volume of atom i. Based on the above

expression, the stress within a specific domain (e.g. PEO/SiO_2 interface and PEO matrix) can be computed by summing up the per-atom contribution of all atoms within the domain and dividing with its volume.

The spatial distribution of strain fields at an atomic level can be probed directly for any system under study. In this approach, the strain distribution is computed by using concepts from continuum mechanics to define the strain per atom, relating the kinematics and the positions of atoms at an initial time t_0 and at a subsequent time t_1. The coordinate vectors of atoms in the initial reference configuration Ω_0 at time t_0 having a Cartesian basis $\tilde{\mathbf{e}}_i$, and in the configuration Ω_0 at time t_1 with a Cartesian basis $\mathbf{e}_i$ are represented by $\mathbf{X}$ and $\mathbf{x}$, respectively (Figure S3). Then the mapping from position vector $\mathbf{X}$ to $\mathbf{x}$, is denoted by $\boldsymbol{\Lambda}$ so that

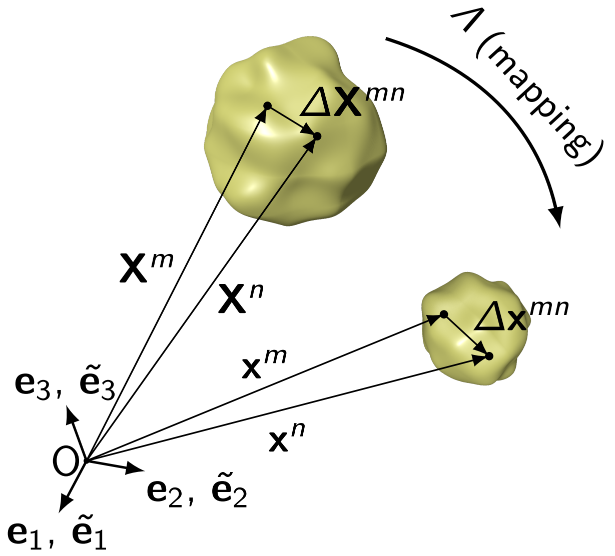

Figure S3: Kinematics of neighboring atoms at different times. A region in space is shown, where $\mathbf{X}$ and $\mathbf{x}$ represent the coordinate vectors of atoms in the reference configuration Ω_0 at time t_0, and in the configuration Ω_1 at time t_1, respectively.

$$\mathbf{X} = X_i\tilde{\mathbf{e}}_i, \quad \mathbf{x} = x_i\mathbf{e}_i, \quad \mathbf{x} = \boldsymbol{\Lambda}(\mathbf{X}), \tag{S2}$$

for some coordinates $\mathbf{X}_i$ and $\mathbf{x}_i$ in their respective basis vectors. Assuming sufficient continuity, the local deformation at the spatial point $\mathbf{X}$ is characterized as the gradient of the mapping

function motion and defined as

$$\mathbf{F} = \boldsymbol{\nabla}_{\mathbf{X}}\boldsymbol{\Lambda} = \boldsymbol{\nabla}_{\mathbf{X}}\mathbf{x}, \tag{S3}$$

where $\boldsymbol{\nabla}_{\mathbf{X}}$ denotes the gradient operator taken in with respect to $\mathbf{X}$. The deformation gradient $\mathbf{F}$ obtained from Eq. (S3) is applicable at atomic scales and is also available for the continuum framework.

It is expected that the deformation in the neighborhood of atom m is characterized by the changes in the relative position of its neighbor n. Atom m is located at position $\mathbf{X}^m$ in the reference configuration Ω_0 and position $\mathbf{x}^m$ in the current configuration Ω_1. Then, the relative position of neighboring atom n and the deformation gradient $\mathbf{F}^m$ at atom m is related to its neighboring atoms, and satisfies

$$\Delta\mathbf{x}^{nm} = \mathbf{F}^m \cdot \Delta\mathbf{X}^{nm}, \tag{S4a}$$

where $\cdot$ denotes dot product tensor contractions and

$$\Delta\mathbf{x}^{nm} = \mathbf{x}^n - \mathbf{x}^m, \tag{S4b}$$

$$\Delta\mathbf{X}^{nm} = \mathbf{X}^n - \mathbf{X}^m. \tag{S4c}$$

In principle, for a given reference atom m, Eq. (S4a) must hold for all its neighboring atoms. However, $\mathbf{F}^m$ of atom m cannot be generally determined from a single atom n, since, unlike the continuum limit, the interparticle interactions with all its neighboring atoms need to be taken into account, as their motion is strongly correlated. Therefore, we seek an optimal local deformation gradient $\hat{\mathbf{F}}^m(\mathbf{x})$, which is obtained from the minimization problem:

$$\hat{\mathbf{F}}^m = \underset{\mathbf{F}^m}{\operatorname{argmin}}\, W^m(\mathbf{x}, \mathbf{X}; \mathbf{F}^m), \tag{S5}$$

i.e. it minimizes the squared error

$$W^m(\mathbf{x}, \mathbf{X}; \mathbf{F}^m) = \sum_{n=1}^{N} \left\| \Delta\mathbf{x}^{nm} - \mathbf{F}^m(x) \cdot \Delta\mathbf{X}^{nm}) \right\|^2 , \tag{S6}$$

where N is the number of neighboring atoms within the cutoff radius r_{cut} and $\| \cdot \|$ denotes the Euclidean norm. From the optimal deformation gradient matrix $\hat{\mathbf{F}}^m(\mathbf{x})$, we can then define the Lagrange Green strain tensor $\boldsymbol{\varepsilon}^m(\mathbf{x})$ with respect to the reference coordinates as:

$$\boldsymbol{\varepsilon}^m = \frac{1}{2} \left[\hat{\mathbf{F}}^m \cdot \left(\hat{\mathbf{F}}^m \right)^T - \mathbf{I} \right] , \tag{S7}$$

where, for each atom m within the simulation box, ε^m is a symmetric 3×3 tensor.

S4 Structure and Dynamics of Polymer Chains in the Interfacial Zone

Regarding the influence of silica nanoparticles on polymer dynamics, in a recent work by our group, we examined the spatial heterogeneous dynamics and glass transition of polymer chains in PEO/SiO_2 nanocomposites with varying silica concentration.[9] The results demonstrate the existence of a spatial gradient in the segmental dynamics and the glass transition temperature, as well. In more detail, a layer-resolved analysis of the mean squared displacement (MSD) of the polymer chains revealed that the polymer chains belonging to the matrix region are more mobile than their counterparts residing in the interphase region as it can be seen in Figure S4 where the MSD of the interphase and matrix regions is plotted as a function of time at temperatures T = 300 and T = 400K.

Focusing on Figure S4, we can also deduce that the slowing down in polyner mobility becomes more pronounced upon approaching the glassy regime (T = 300K). This implies that confinement effects have a higher impact on the polymer dynamics in the glassy state.

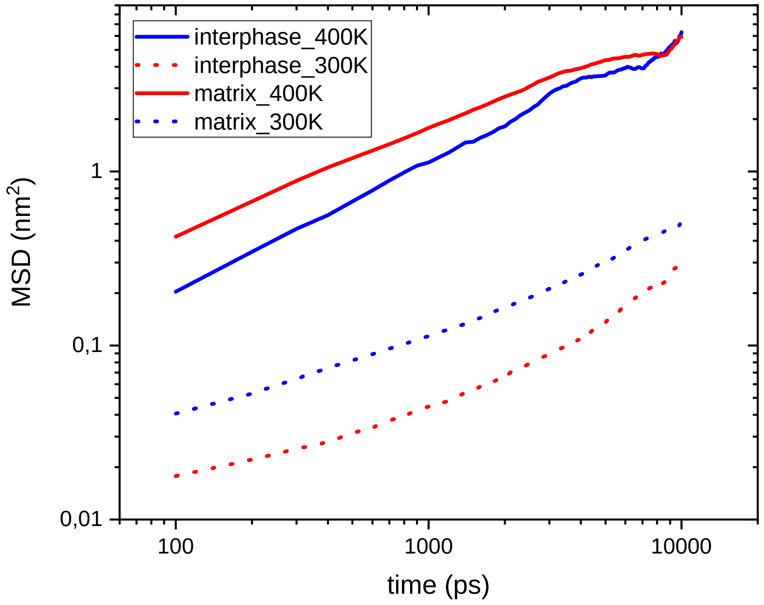

Figure S4: Mean squared displacement (MSD) of the polymer chains residing at the interphase or matrix regions at T= 300, 400 K. .

The end-to-end distance, R_e, and the radius of gyration, R_g, of the PEO chains are calculated as a function of the distance of their center of mass from the NP surface at T=400 K. For this, the region around the NP's is divided into spherical shells with a thickness of 6 Å). R_e and R_g are averaged over all free polymer chains in the PNC. As shown in Figure S5 at larger distances from the NP surface (i.e. 10 Å),R_e and R_g of the polymer chains in all systems converge to the corresponding values of the homogeneous (pure) polymer.[10] It is clear that for the current systems the nanofiller does not influence significantly the structural properties of the bulk polymer chains beyond a distance of about 10 Åfrom the surface of the NP.

S5 Determination of glass transition temperature

The transition temperature is a critical property in our study. Actually, the glass transition temperature of the examined system has already been calculated recently by our group.[9] Figure S6 illustrates the temperature dependence of the specific volume of the nanocomposite

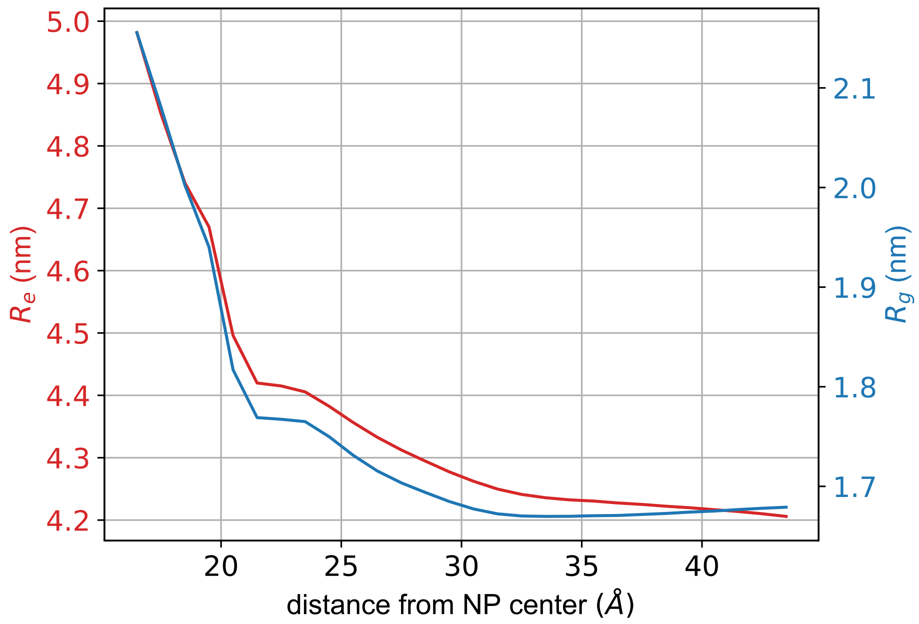

Figure S5: End-to-end distances (Re) and the radius of gyration (Rg) of the polymer chains as a function of the distance of their center of mass from the nanoparticleat T= 400 K. .

PEImodel. The T_g value, as extracted from the intersection of the linear fits to the two regimes of distinct slope is 285K.[9]

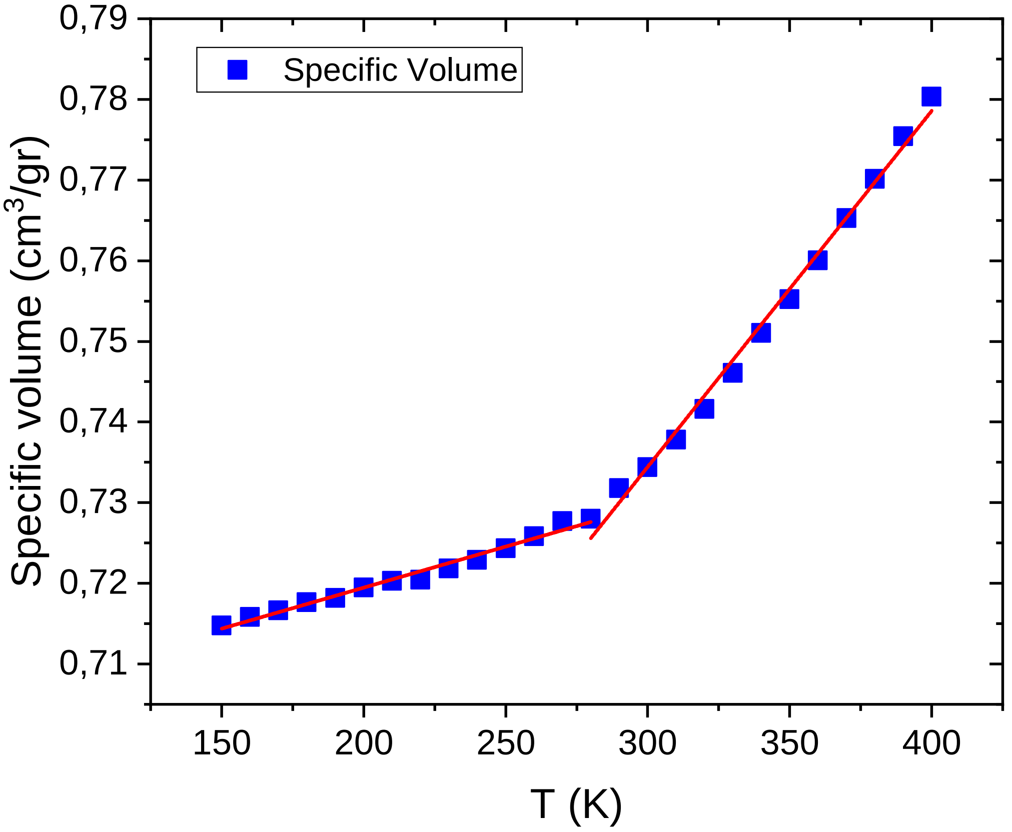

Figure S6: Temperature dependence of the specific volume of the nanocomposite model. The red lines denote the linear fits to the glassy and melt regimes.

References

(1) Hong, B.; Panagiotopoulos, A. Z. Molecular Dynamics Simulations of Silica Nanoparticles Grafted with Poly(ethylene oxide) Oligomer Chains. *The Journal of Physical Chemistry B* **2012**, *116*, 2385–2395, PMID: 22243140.

(2) Fischer, F.; Waitz, T.; Vollath, D.; Simha, N. On the role of surface energy and surface stress in phase-transforming nanoparticles. *Progress in Materials Science* **2008**, *53*, 481–527.

(3) Pandey, Y.; Doxastakis, M. Detailed atomistic Monte Carlo simulations of a polymer melt on a solid surface and around a nanoparticle. *The Journal of chemical physics* **2012**, *136 9*, 094901.

(4) Darden, T.; York, D.; Pedersen, L. Particle mesh Ewald: An $N \cdot \log(N)$ method for Ewald sums in large systems. *The Journal of Chemical Physics* **1993**, *98*, 10089–10092.

(5) Parrinello, M.; Rahman, A. Polymorphic transitions in single crystals: A new molecular dynamics method. *Journal of Applied Physics* **1981**, *52*, 7182–7190.

(6) Hoover, W. G. Canonical dynamics: Equilibrium phase-space distributions. *Phys. Rev. A* **1985**, *31*, 1695–1697.

(7) Thompson, A. P.; Aktulga, H. M.; Berger, R.; Bolintineanu, D. S.; Brown, W. M.; Crozier, P. S.; in 't Veld, P. J.; Kohlmeyer, A.; Moore, S. G.; Nguyen, T. D.; et al., LAMMPS - a flexible simulation tool for particle-based materials modeling at the atomic, meso, and continuum scales. *Computer Physics Communications* **2022**, *271*, 108171.

(8) Kawaguchi, S.; Imai, G.; Suzuki, J.; Miyahara, A.; Kitano, T.; Ito, K. Aqueous solution properties of oligo- and poly(ethylene oxide) by static light scattering and intrinsic viscosity. *Polymer* **1997**, *38*, 2885–2891.

(9) Tanis, I.; Power, A. J.; Chazirakis, A.; Harmandaris, V. A. Heterogeneous Glass Transition Behavior of Poly(Ethylene oxide)/Silica Nanocomposites via Atomistic MD Simulations. *Macromolecules* **2023**, *56*, 5482–5489.

(10) Power, A. J.; Papananou, H.; Rissanou, A. N.; Labardi, M.; Chrissopoulou, K.; Harmandaris, V.; Anastasiadis, S. H. Dynamics of Polymer Chains in Poly(ethylene oxide)/Silica Nanocomposites via a Combined Computational and Experimental Approach. *The Journal of Physical Chemistry B* **2022**, *126*, 7745–7760.
